# A twin xanthan lyase-dependent xanthan degradation system in *Paenibacillus taichungensis* I5

**DOI:** 10.1007/s00253-025-13684-y

**Published:** 2026-01-22

**Authors:** Rui Han, Melanie Baudrexl, Oliver Frank, Christina Ludwig, Oksana V. Berezina, Sergey V. Rykov, Wolfgang Liebl

**Affiliations:** 1 Chair of Microbiology, School of Life Sciences, Technical University of Munich, Emil-Ramann-Str. 4, 85354 Freising, Germany; 2 Michael Smith Laboratories, University of British Columbia, 2185 East Mall, Vancouver, BC V6T 1Z4 Canada; 3 Chair of Food Chemistry and Molecular Sensory Science, School of Life Sciences, Technical University of Munich, Lise-Meitner-Straße 34, 85354 Freising, Germany; 4 Bavarian Center for Biomolecular Mass Spectrometry (BayBioMS), School of Life Sciences, Technical University of Munich, Gregor-Mendel-Str. 4, 85354 Freising, Germany; 5 National Research Centre “Kurchatov Institute”, Academician Kurchatov Sq. 1, 123182 Moscow, Russian Federation

**Keywords:** *Paenibacillus taichungensis*, Xanthan gum, Gene cluster, Xanthanase, Xanthan lyase

## Abstract

**Abstract:**

Xanthan gum, a natural heteropolysaccharide produced by *Xanthomonas* species*,* has many biotechnological applications across industries due to its unique rheological properties. Expanding its utility requires specific enzymes capable of targeted xanthan modification or degradation. In this study, a novel bacterial strain, isolated from a spoiled xanthan sample and identified as *Paenibacillus taichungensis* I5, was shown to degrade xanthan using a plate screening assay with Congo red. Activity tests of crude enzyme in culture supernatant demonstrated the secretion of xanthan-degrading enzymes. Genome and proteome analyses suggest a chromosomal xanthan utilization locus encoding a suite of enzymes, including a xanthanase (Pt_XanGH9), two xanthan lyases (Pt_XanPL8a and Pt_XanPL8b), two unsaturated glucuronidases, two α-mannosidases, as well as transport and regulator proteins. Functional characterization through recombinant protein expression and enzyme assays confirmed the functions of Pt_XanGH9, Pt_XanPL8a and Pt_XanPL8b on native xanthan and xanthan-derived oligosaccharides. The polysaccharide degradation products released by these enzymes were identified via LC–MS analysis and suggested two xanthan lyases with divergent cleavage preferences. In contrast to Pt_XanPL8a, Pt_XanPL8b is synthesized with an N-terminal signal peptide, yet both lyases were detected in cell-free supernatant during growth on xanthan. Based on the composition of the xanthan utilization gene cluster and preliminary enzyme characteristics, a working model for xanthan utilization by *P. taichungensis* I5 is proposed. Reaching a better understanding of bacterial xanthan degrading pathways and the enzymes involved may help to develop modified xanthan derivatives and xanthan degrading enzymes that align with the specific demands of various industrial process.

**Key points:**

• *The genome of P. taichungensis I5 encodes a xanthan utilization locus.*

• *P. taichungensis I5 employs a twin lyase-dependent xanthan utilization system.*

• *The two xanthan lyases differ in cellular localization and in cleavage specificity.*

**Supplementary Information:**

The online version contains supplementary material available at 10.1007/s00253-025-13684-y.

## Introduction

The bacteria belonging to the *Paenibacillus* genus were originally classified as representatives of the genus *Bacillus* and reassigned to a new genus in 1993 (Ash et al. 1993). *Paenibacillus* species have been identified in various habitats, with many species found in association with humans, animals, and/or plants. For example, *Paenibacillus polymyxa* is a common species found in the rhizosphere of different crops (Daud et al. 2019), while *Paenibacillus* *vulneris* was isolated from a necrotic wound of a patient (Glaeser et al. 2013). The *Paenibacillus* species are physiologically versatile facultative anaerobes that are able to form endospores, thereby allowing them to survive in harsh conditions such as Antarctic sediment (Montes et al. 2004) and desert soil (Lim et al. 2006). Notably, they have great capacities to produce a wide range of natural products, including exo-polysaccharides, extracellular enzymes such as proteases (von der Weid et al. 2003), lipases (Cao et al. 2015), cellulases, xylanases (Rivas et al. 2006), amylases (Uozumi et al. 1989) as well as a number of antibiotics (DeCrescenzo Henriksen et al. 2007; Martin et al. 2003). This potential makes them a promising source of enzymes for applications in the food, feed, textile, and biofuel industries and in healthcare (Padda et al. 2017; Yang et al. 2016). Metabolic pathways of *Paenibacillus* representatives support the efficient metabolism of a variety of plant-derived polysaccharides as carbon sources, e.g. cellulose, hemicellulose and pectin (Li et al. 2023). Additionally, genomic analysis has revealed the presence of a broad repertoire of glycoside hydrolases (GH), which likely provide a competitive advantage in adapting to complex and variable environments (Phakeenuya et al. 2020).

Xanthan has a β−1,4-linked cellulose-like backbone, which is heavily decorated with trisaccharide side chains on every second glucose residue comprising mannose linked β−1,4-glycosidically to glucuronic acid linked in β−1,2-fashion to mannose linked in α−1,3-mode to the glucose backbone moiety. The side chains feature some compositional variability, with decorations of acetyl groups and pyruvyl groups at various positions, although acetylation is often located in the inner mannose and pyruvylation tends to be present at the outer mannose (Kool et al. 2014). Xanthan displays a double-stranded helix structure in solution, while undergoing order–disorder conformational transitions with changes in temperature and salt (Brunchi et al. 2019). These properties make it highly resistant to degradation by many microorganisms. However, certain bacteria have developed specialized utilization systems capable of degrading xanthan into smaller, more readily usable sugars. The enzymatic breakdown of xanthan is a complex process that requires collaboration of a set of enzymes, including xanthan lyase, xanthanase (or β-d-glucanase), β-d-glucosidase, β-d-glucuronidase and α-d-mannosidase, which were first reported in strain *Bacillus* sp. GL1 (Nankai et al. 1999). This lyase-dependent xanthan degradation pathway initially starts with the cleavage of xanthan side chains by xanthan lyase, which removes the terminal (pyruvylated) mannose from the side chains via β-elimination, thereby facilitating the access by xanthanases (or β-d-glucanases) to break down the β-d-glucan backbone. Subsequently, xanthan tetrasaccharides (unsaturated glucuronyl-acetylated mannosyl-glucosyl-glucose) are extracellularly formed and transported into the cell followed by further degradation by intracellular enzymes. Most of reported strains, e.g. *Microbacterium* sp. XT11 (Sun et al. 2019), gut bacteria *Ruminococcaceae* UCG-13 and *Bacteroides intestinalis* (Ostrowski et al. 2022), are consistent with the lyase-dependent xanthan degradation pathway (Berezina et al. 2024). However, the thermophilic planctomycete *Thermogutta terrifontis* was suggested to employ a lyase-independent system due to the absence of potential xanthan lyase in the genomic and transcriptomic analyses (Elcheninov et al. 2017).

In this work, we introduce a novel xanthan degrader, *Paenibacillus taichungensis* I5, which was isolated from a spoiled xanthan sample. To investigate its capacity for xanthan decomposition, we developed a plate screening assay with Congo red and used thin layer chromatography (TLC) to detect breakdown products. Using genomic and proteomic analysis of strain I5 followed by heterologous enzyme production in *Escherichia coli* and enzyme activity assays with a xanthanase and two xanthan lyase enzyme candidates, a novel twin lyase-dependent xanthan degradation system can be proposed. Observed for the first time in strain *P.* *taichungensis* I5, this system provides further insights into bacterial metabolic strategies for utilization of the complex polysaccharide xanthan.

## Material and methods

### Identification of a new xanthan degrader

A bacterial strain was isolated from a spoiled xanthan-based thickener obtained from a food production plant in St. Petersburg, Russia. The xanthan-degrading capability of the isolate was visualized using a plate screening assay with Congo red. To this end, xanthan mineral medium (XMM) containing xanthan gum (LANUCO GmbH, Hamburg, Germany) as the sole carbon source was used as described in a previously reported protocol (Han et al. 2024). Briefly, a thin layer (approximately 1.5–2 mm) of XMM containing 0.05% xanthan and 2% agar was poured into sterile Petri dishes after autoclaving. Fresh overnight cultures of strain I5 and *E. coli* DSM 30083^T^ grown in Lysogeny Broth (LB) medium at 30 °C were spotted on the surface of the XMM agar plates. Following an incubation at 30 °C for 24 h, the plate was stained with a 2% Congo red solution for 10 min, and then washed twice with a 1 M NaCl solution for 10 min each. With the plate assay, hydrolysis of xanthan is visualized by the halo formation around the colonies.

To access the hydrolytic activities of the extracellular enzymes produced by the strain, cultures were grown in 100 mL Erlenmeyer flasks containing XMM liquid medium at 30 °C, 180 rpm. Following a 20 h-incubation, the cell-free supernatant containing secreted enzymes was collected by centrifugation at 4 °C, 8,000 rpm for 10 min. An equal volume of fresh XMM medium was then mixed with the collected supernatant and incubated at 37 °C, 180 rpm for 24 h to allow enzymatic depolymerization of xanthan. After incubation, the reaction mixture was analyzed by thin-layer chromatography (TLC). Aliquots of the supernatant were spotted on TLC plates (Silica gel 60, 20 cm × 20 cm, Merck, Darmstadt, Germany). Additionally, 1 µg samples of mixtures of cellodextrins (glucose, cellobiose, cellotriose, cellotetraose, cellopentaose and cellohexaose) and xylooligosaccharides (xylose, xylobiose, xylotriose, xylotetraose, xylopentaose and xylohexaose) were used as reference substances. After chromatographic separation using a mixture of butanol, ethanol and water (5: 5: 4, v/v/v), the dried plate was sprayed with staining reagent (9 mL of 100 mL acetone containing 1 g diphenylamine and 1 mL aniline, mixed with 1 mL of ~ 85% phosphoric acid) (Leis et al. 2015). The detection of degradation products was achieved by heating the TLC plate on a metal plate laying on a magnetic stirrer set to 120 °C.

### Genome and proteome analysis

The strain shares almost 100% 16S rDNA sequence identity with *P. taichungensis* and has been deposited in the All-Russian Collection of Microorganisms (VKM) as *P. taichungensis* I5 (VKM B-3510D) (Denisenko et al. 2021). Genomic DNA was extracted and sequenced at the ZIEL Core Facility Microbiome (TU Munich, Freising, Germany) by Illumina PE150 (San Diego, CA, USA) shotgun sequencing as described before (Han et al. 2024). Open reading frames (ORFs) were annotated using Prokka v.1.14.6 (Seemann 2014) and ORFs resembling carbohydrate-active enzymes (CAZymes) were subsequently annotated using the dbCAN2 database (Zhang et al. 2018). To determine taxonomic relatedness, overall genome relatedness indices (OGRI) were calculated, including digital DNA–DNA hybridization (dDDH) via the Genome-to-Genome Distance Calculator (GGDC) (https://ggdc.dsmz.de/) and average nucleotide identity (ANI) values using OrthANIu (www.ezbiocloud.net/tools/ani). In addition, a pan-genome analysis was conducted using the Bacterial Pan Genome Analysis Pipeline (BPGA) (Chaudhari et al. 2016), incorporating 13 additional *P. taichungensis* genomes from the NCBI database. Orthologous gene clustering was performed using the USEARCH algorithm with a 90% similarity threshold. Functional categorization of gene clusters was carried out using the COG and KEGG databases. Core genes were defined as those present in all 14 genomes, unique genes as those present exclusively in one genome, and accessory genes as those present in 2 to 13 genomes. Genes present in one or more genomes but completely absent in the current genomes under analysis were referred to as 'exclusively absent genes'.

For secretome analysis, *P. taichungensis* strain I5 was cultured at 30 °C, 180 rpm in triplicates under three different conditions: mineral medium supplemented with either 0.5% (w/v) xanthan or glucose as the sole carbon source, and LB complex medium. Cell-free culture supernatants (2 mL) were harvested in the late exponential growth phase by centrifugation. Three biological replicates of 25 μL culture supernatant were each mixed with 10 µL of LDS sample buffer (ThermoFisher Scientific, Waltham, MA, USA) and heated at 95 °C for 10 min. Proteins in the samples were subjected to in-gel trypsin digestion, measured by liquid chromatography-tandem mass spectrometry (LC–MS/MS) and analyzed with the software tool MaxQuant as previously described (Han et al. 2024). The mass spectrometric raw files as well as the MaxQuant output files have been deposited to the ProteomeXchange Consortium via the PRIDE partner repository and can be accessed using the identifier PXD068698.

### Gene manipulations and enzyme production

To investigate the function of the genes clustered in the xanthan utilization locus, genes encoding the putative xanthanase and xanthan lyases were initially amplified using Q5-polymerase according to the manufacturer’s protocol (New England Biolabs, Feldkirchen, Germany). Primers used for gene amplification can be found in supplementary Table S1. The amplicons were cloned into *Nde*I/*Xho*I-linearized pET24c expression vector (Merck, Darmstadt, Germany) via Gibson Assembly (New England Biolabs, Frankfurt, Germany), followed by introduction into *E. coli* DH10B competent cells (New England Biolabs, Frankfurt, Germany) by heat-shock transformation. After isolation of recombinant plasmids from transformants on LB plates containing 50 μg mL^−1^ kanamycin by using the Monarch® Plasmid Miniprep Kit (New England BioLabs), the correctness of these plasmids was verified by sequence analysis (Eurofins Genomics, Ebersberg, Germany).

The constructed plasmids were transferred into *E. coli* Rosetta™ 2 (Merck, Darmstadt, Germany) by electroporation. Single colonies grown on agar plates containing 50 μg mL^−1^ kanamycin and 34 μg mL^−1^ chloramphenicol were inoculated in fresh LB medium containing both antibiotics. When the OD_600_ of the cell culture reached 0.5 after around 2 h growth at 37 °C, 0.5 mM isopropyl-β-D-1-thiogalactopyranoside (IPTG) was added to induce enzyme production at 16 °C, 180 rpm. The cells were harvested after 24 h by centrifugation (4 °C, 12,000 × *g*, 30 min) and then stored at −20 °C until further study. Protein purification using HisTrap FF columns (Cytiva Europe GmbH, Freiburg, Germany) was performed as previously described (Han et al. 2024). Subsequently, enzyme production was confirmed by SDS-PAGE and western-blot, where Anti-His (C-term)/AP Ab (1: 3000, Thermo Fisher Scientific, Waltham, MA, USA) and Novex™ AP Chromogenic Substrate (BCIP/NBT, Thermo Fisher Scientific, Waltham, MA, USA) were used for the visualization of fusion proteins with His-tag.

### Enzymatic activity and xanthan oligosaccharide purification

To determine the xanthan-degrading activity, ‘native xanthan’ (LANUCO GmbH, Hamburg, Germany) was used as the substrate. 250 mg xanthan powder were dissolved in 25 mL Milli-Q water (ELGA LabWater, High Wycombe, UK) by constantly stirring. 100 μL enzyme reactions in duplicates containing 50 μL xanthan solution, 10 μL 0.5 M HEPES–NaOH (pH 7.0) and recombinant enzyme (xanthanase Pt_XanGH9, xanthan lyase Pt_XanPL8a, xanthan lyase Pt_XanPL8b) were incubated at 37 °C for 24 h. Reactions were stopped by heating to 95 °C for 10 min. After centrifugation, the reducing sugars released by the enzymes were determined with the 3,5-dinitrosalicylic acid (DNSA) assay and absorbance measurement at 540 nm (Jain et al. 2020), using glucose as standard.

To investigate the cleavage specificity of the *P. taichungensis* I5 xanthan lyases, pentasaccharides (the repeating units of xanthan released by xanthanase Pt_XanGH9 in this study) were collected from enzyme reaction mixtures by centrifugation using 10 K MWCO concentrators (Vivaspin® Turbo 20, Sartorius AG, Goettingen, Germany). The purification of xanthan oligosaccharides was achieved on a preparative liquid chromatography system (SpotPrep, Gilson, Berlin, Germany) equipped with a NUCLEODUR HILIC column (21 mm × 250 mm, 5 μm, Macherey–Nagel, Dueren, Germany) and an evaporative light scattering detector SEDEX 85 (Knauer, Berlin, Germany). The program of the chromatography system was executed with the following conditions and settings, using 10 mM ammonium acetate and a decreasing acetonitrile gradient as eluent: 0–2.35 min, 95% acetonitrile; 2.35–22.35 min, a linear 95% to 20% acetonitrile gradient, followed by constant 20% acetonitrile for 5 min; 27.35–30.35 min, 20% to 95% linear acetonitrile gradient, followed by constant 95% acetonitrile for 2 min. The flow rate was set at 21 mL min^−1^. The eluate from the column was split at a ratio of 1: 20 and monitored in parallel with both an evaporative light scattering detector (ELSD) and a UV/Vis detector (220 nm). The peaks representing potential oligosaccharides were further identified by mass spectrometry (MS) analysis as described below. The successfully purified pentasaccharides were dried by vacuum rotary evaporation and dissolved in Milli-Q water for use as substrate to investigate xanthan lyase activity.

### TOF–MS analysis of xanthan degradation products

For analysis of products released by enzymatic cleavage of xanthan or xanthan oligosaccharides by the xanthanase Pt_XanGH9 and two xanthan lyases Pt_XanPL8a and Pt_XanPL8b, a Waters Synapt G2-S HDMS mass spectrometer (Waters, Eschborn, Germany) operating in the negative electrospray ionization (ESI^−^) mode in combination with an Acquity UPLC core system (Waters, Eschborn, Germany) for chromatographic separation, which was equipped with a sample manager, binary pump manager, and column over (45 °C), was used. The samples were injected onto an Acquity BEH C18 column (150 × 2.1 mm, 1.7 μm, 130 Å; Waters, Eschborn, Germany). A flow rate of 0.4 mL min^−1^ was used, with formic acid (0.1%) as the aqueous solvent and acetonitrile as the organic solvent. For separation of the analytes, an acetonitrile gradient started at 1% and was gradually increased to 100% over four minutes and maintained for 30 s. Data processing was performed using the MassLynx V4.1 software (Waters, Eschborn, Germany).

## Results

### Demonstration of xanthan-degrading capacity

**Fig. 1:**
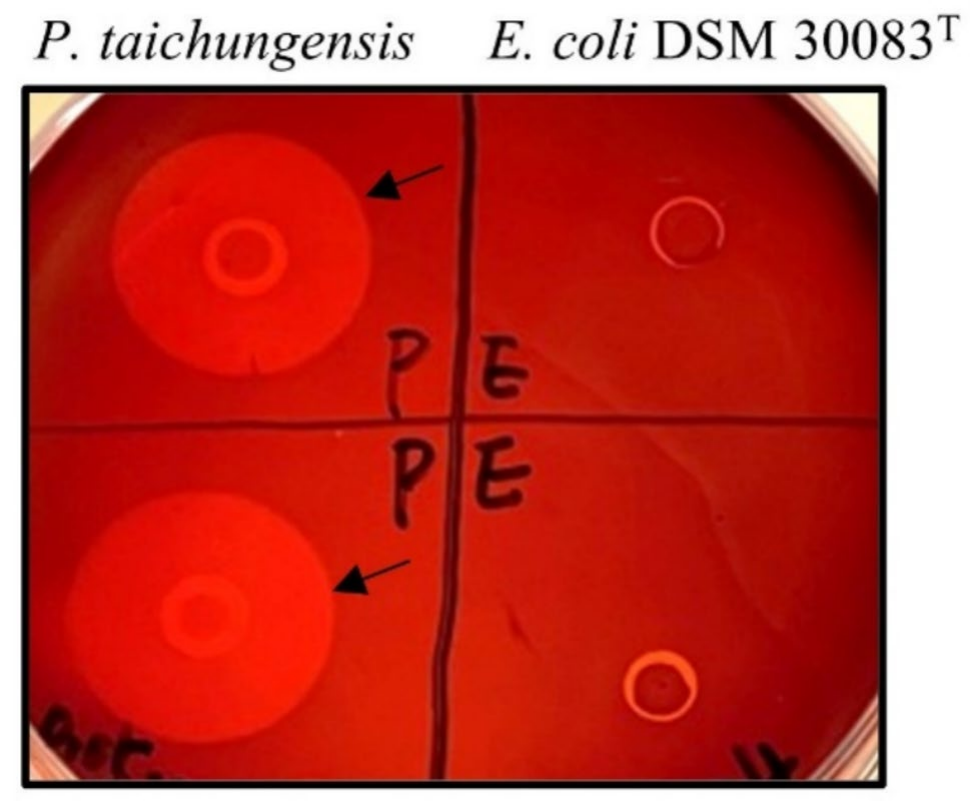
Xanthan degradation plate assay. Demonstration of bacterial xanthan-degrading capacity by Congo red assay on agar plate with 0.05% xanthan as a sole carbon source. Letters ‘P’ and ‘E’ on the plate represent *P. taichungensis* I5 and *E. coli* DSM 30083^ T^, respectively. Halo formation indicates xanthan degradation by bacterial growth on the plate

The strain *P.* *taichungensis* I5, isolated from a spoiled xanthan sample, is able to liquefy xanthan dissolved in the growth medium (Denisenko et al. 2021), which indicates its capacity to secrete xanthan-degrading enzymes. In this study, its xanthan-degrading ability was demonstrated using a plate screening assay based on polysaccharide staining with Congo red. Samples of freshly grown *P.* *taichungensis* I5 and *E. coli* strain DSM 30083^T^ were spotted on the surface of xanthan mineral medium (XMM) plate and incubated at 30 °C for 24 h. While strain *P.* *taichungensis* I5 grew well with xanthan as the sole carbon source, *E. coli* could not grow due to its inability to utilize xanthan. Following staining with 2% Congo red solution and washing with 1 M NaCl, clear halos were observed in the vicinity of *P.* *taichungensis* I5 colonies (see arrows in Fig. 1), which points to the production of xanthan-degrading enzymes by this strain.

To further study the xanthan degrading activity of extracellular enzymes secreted by *P.* *taichungensis* I5, cell-free supernatant from the culture grown in XMM was collected by centrifugation and incubated with an equal volume of sterile XMM. After incubation, 1 μL, 2 μL and 6 μL of supernatant were spotted on a silica gel 60 TLC plate for product separation in the mobile phase. Staining with an acetone solution containing diphenylamine, aniline and phosphoric acid revealed a series of spots on the plate (see supplementary Fig. S1a), which indicates that the enzymes released into the supernatant during bacterial growth on xanthan-containing medium can depolymerize xanthan into monosaccharides and various oligosaccharides. It cannot be excluded, however, that also intracellular enzymes were released to the culture supernatant e.g. by lysis of some cells. Due to the lack of suited oligosaccharide standards, the precise chemical nature of the xanthan degradation products could not be identified by TLC. Besides, no product spots were detected after TLC of supernatant samples from the spent XMM culture (SN_pt_ in supplementary Fig. S1a), suggesting that during growth on xanthan all released oligo- and monosaccharides were metabolized by the cells and that there was no accumulation of xanthan breakdown products in the culture medium.

### Genomic features of strain *P. taichungensis* I5

**Fig. 2:**
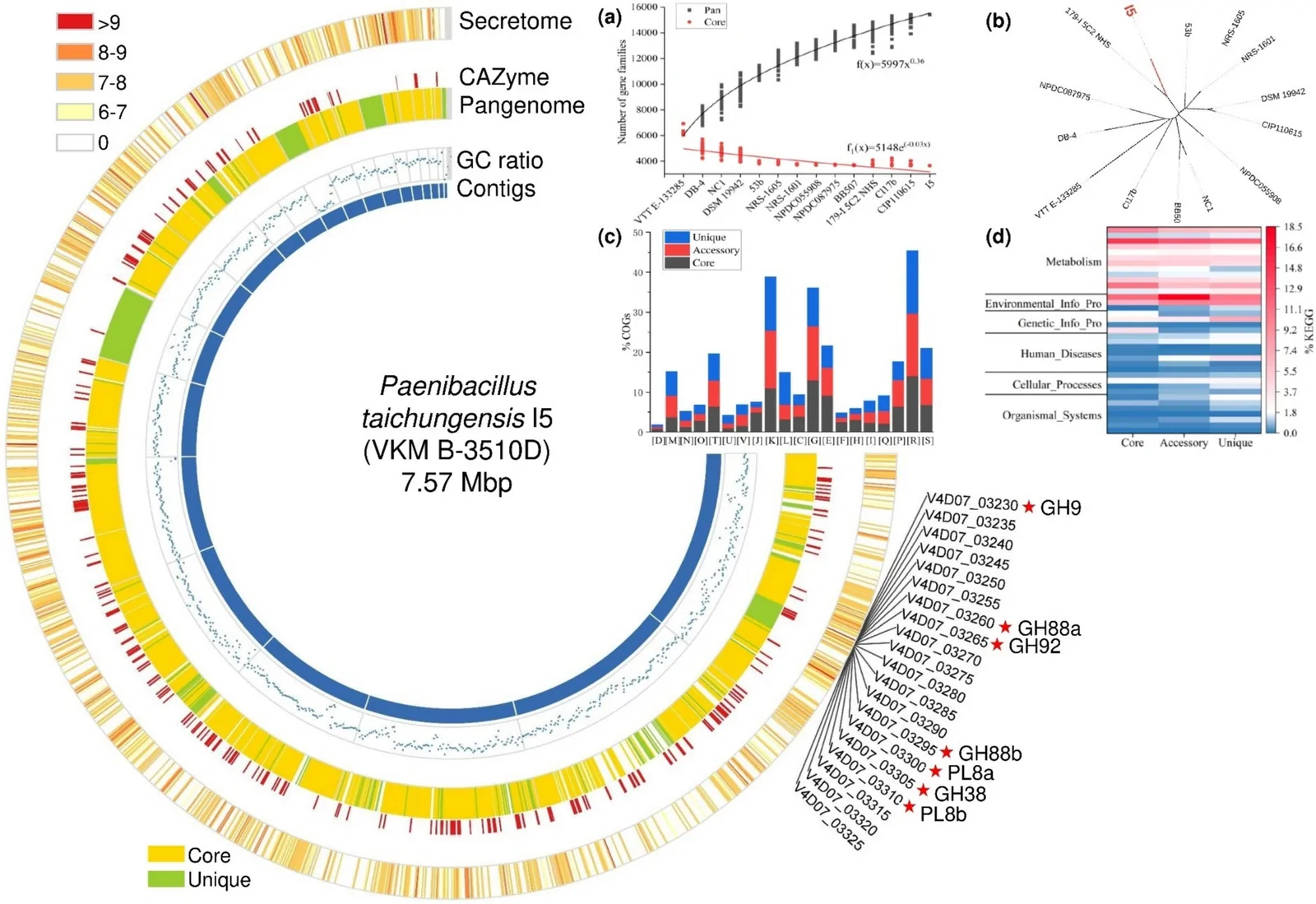
Genome analysis of *P. taichungensis* I5 VKM B-3510D and pan-genome analysis of available genomes of the species *P. taichungensis*. The plot in the center represents the genome of *P. taichungensis* I5 VKM B-3510D consisting of 29 contigs (inner, blue layer, ordered clockwise by decreasing contig size). From the innermost to the outermost concentric layers, the plot shows general information including the GC ratio (32.58%–54.42%), the core and unique genes identified through pan-genome analysis using BPGA (see supplementary Table S3), the CAZyme families predicted by dbCAN2, and the bacterial secretome pattern (outermost circle), i.e. the proteins identified by LC–MS/MS in the culture supernatant when strain I5 grew in xanthan-containing XMM medium with the color distribution indicated in the upper left corner. The values determining the color shades were derived from the measured mass spectrometric protein intensities across three biological replicates. A log₁₀ transformation was applied for the total iBAQ values. The genes marked with NCBI locus tags and red stars are located in a xanthan utilization gene cluster. Small figure insets in the top right corner provide information about **a** cumulative total and core gene families with increasing number of *P. taichungensis* genomes in a pan-core gene plot with corresponding fitting curves; **b** phylogenetic analysis of the *P. taichungensis* pan-genome from 14 strains; **c** COG and **d** KEGG distribution of core, accessory and unique genes. Detailed COGs categories include: C, energy production and conversion; D, cell cycle control, cell division and chromosome partitioning; E, amino acid transport and metabolism; F, nucleotide transport and metabolism; G, carbohydrate transport and metabolism; H, coenzyme transport and metabolism; I, lipid transport and metabolism; J, translation, ribosomal structure and biogenesis; K, transcription; L, replication, recombination and repair; M, cell wall/membrane/envelop biogenesis; N, cell motility; O, post-translational modification, protein turnover, chaperones; P, inorganic ion transport and metabolism; Q, secondary metabolites biosynthesis, transport and catabolism; R, general functional prediction only; S, function unknown; T, signal transduction mechanisms; U, intracellular trafficking, secretion and vesicular transport; V, defense mechanisms. The third line in (d) KEGG analysis, all marked in red, represents carbohydrate metabolism. Other detailed annotations can be found in the supplementary material (Table S5 and Table S6)

The whole genome sequence of *P. taichungensis* strain I5, which was determined with Illumina sequencing technology and comprised 29 contigs, has been submitted to the NCBI database with the accession number JAZHOZ000000000. The draft genome size of *P. taichungensis* I5 was determined as 7.57 Mbp (Fig. 2) with a GC content of 45.53%. The genome sequence was compared with 13 other *P.* *taichungensis* genomes publicly available from the NCBI database. As shown in supplementary Fig. S2, ANI and dDDH values of about 94–99% and 56–88%, respectively (with the exception of strain VTT E-133285 which appears to be more distantly related), were close to the widely accepted species thresholds of 95%–96% and 70%, respectively. The genome analysis in addition to the almost 100% 16S rDNA sequence identity with the *P. taichungensis* type strain (Denisenko et al. 2021) thus supports the affiliation of strain I5 to the species *P. taichungensis*.

**Fig. 3:**
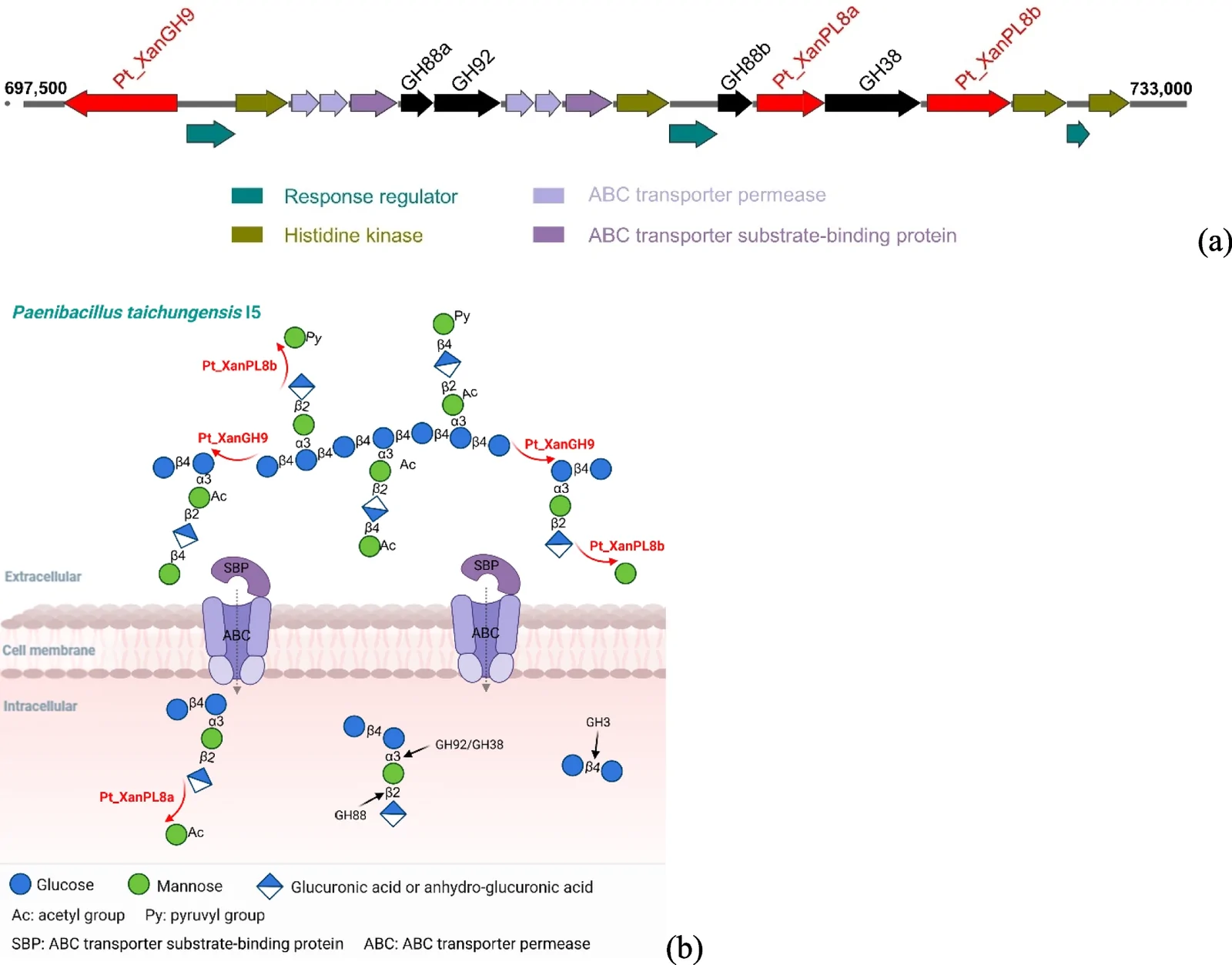
Xanthan utilization gene cluster in *P. taichungensis* I5. **a** The gene cluster has a size of approximately 35.5 kbp and encompasses ORFs for one GH9 (Pt_XanGH9), one GH38, one GH92, two GH88, and two PL8 enzymes (Pt_XanPL8a and Pt_XanPL8b), which collectively represent seven potential xanthan-degrading enzymes. Proteins marked in red were initially characterized in this study. Three arrows drawn below the line of most ORFs represent ORFs with short overlaps of 8–35 nucleotides. All CAZymes were detected in xanthan mineral medium supernatant during the bacterial growth. Other ORFs for putative accessory proteins are marked in different colors based on their functions. More related information can be found in supplementary Table S2. **b** Suggested xanthan degradation pathway in strain I5. Proteins marked in red were characterized while ones in black were identified based on the genome and proteome analysis

Functional annotation by Prokka (Seemann 2014) and dbCAN2 (Zhang et al. 2018) indicated that among 7,003 protein-encoding genes, 275 genes putatively encode carbohydrate-active enzymes (CAZymes), including 184 glycoside hydrolases (GHs), 45 glycosyltransferases (GTs), 13 polysaccharide lyases (PLs) and 22 carbohydrate esterases (CEs). Two of these possible CAZymes from *P.* *taichungensis* I5 (see supplementary Table S2), V4D07_03230 and V4D07_03310, had a high degree of sequence identity over their whole sequence length with a GH9 protein (98.68%) and a PL8 protein (100%), respectively, from *Paenibacillus sonchi*. Closer inspection of the genomic surroundings of these ORFs revealed the presence of a large putative xanthan utilization gene cluster with a length of 35,148 bp in the *P.* *taichungensis* I5 genome (Fig. 3a). This gene cluster contains putative ORFs encoding one glycoside hydrolase family 9 xanthanase (Pt_XanGH9), two mannosidases from GH38 and GH92, two GH88 unsaturated glucuronyl hydrolases, two xanthan lyases from polysaccharide lyase family 8 (PL8) (Pt_XanPL8a and Pt_XanPL8b), and other accessory proteins that may function as transporters of xanthan-derived oligosaccharides and regulators. Among these, two CAZymes (Pt_XanGH9 and Pt_XanPL8b) were suggested to be extracellular enzymes because their ORFs encode secretory precursors with N-terminal signal peptides, while the other five CAZymes are expected to function intracellularly due to the absence of signal peptides. In light of the reported xanthan depolymerization cascade from *Bacillus* sp. GL1, which comprised xanthan lyase, β-d-glucanase (xanthanase), β-d-glucosidase, unsaturated glucuronyl hydrolase and α-d-mannosidase (Nankai et al. 1999), it seems plausible that the related enzymes from *P. taichungensis* I5 may perform similar functions in the xanthan degradation process.

To compare the strain I5 genome with the genomes of closely related bacteria, a pan-genome analysis of the *P. taichungensis* species was conducted, including strain I5 and 13 other strains available as of March 2025 from the NCBI database using BPGA (Chaudhari et al. 2016). The genome sizes of the 14 *P. taichungensis* strains range from 6.89 Mbp (strain 53b) to 7.57 Mbp (strain I5 in this study), corresponding to around 6,000–7,000 proteins encoded in the genomes (see Table 1). All *P. taichungensis* strains have similar genomic GC contents of around 45% although they were isolated from vastly different environments, including forest soil, wolf gut, gold mine, cleanroom floor, freshwater sediment, radioactive repository, pine rhizosphere and spoiled xanthan. Figure 2 depicts some information about the genome of *P.* *taichungensis* I5 (VKM B-3510D), the proteins identified by LC–MS/MS in the culture supernatant when strain I5 grew in xanthan-containing XMM medium (outermost circle in Fig. 2), and statistical data obtained by pan-genome analysis of 14 strains of the species *P.* *taichungensis*. Core genes of the pan-genome are defined as those present in all genomes, while unique genes are those present in only one genome, and accessory genes are those present in more than two genomes but not in the core genes. The pan-genome of the 14 *P. taichungensis* strains consisted of 15,416 gene families, of which 3,655 core genes (in yellow) were shared by all strains, suggesting a high genetic diversity among these bacteria (see supplementary Table S3). With an increasing number of genomes, the pan-gene size increased, whereas the number of core-genes decreased correspondingly (Fig. 2a). The power-law fitting curve indicated an open pan-genome of the *P. taichungensis* species due to the exponent of 0.36. Besides, 2,286 accessory genes from strain I5 were present in 2 to 13 other *P. taichungensis* strains, while 974 unique genes (marked in green in the third concentric layer in Fig. 2) were exclusively present in strain I5. In addition, functional characterization was performed for core, accessory and unique genes using the COG and KEGG databases. As shown in Fig. 2c, the dominant categories of gene functions were R (general function prediction only), K (transcription) and G (carbohydrate transport and metabolism) based on COG annotation. Analysis using the KEGG database also revealed major difference in the metabolism (carbohydrate metabolism) as well as environmental information processing (membrane transport). An unrooted phylogenetic tree (Fig. 2b) shows the relationships between 14 strains based on pan-genome analysis. Strain I5 was closely related to strain 179-I 5C2 NHS (which shows the highest ANI and dDDH values in supplementary Fig. S2) in the same clade, but was separated from the genome of other strains. Given the isolation of these strains from disparate habitats, including soil, gold mine, sediment and disposal (see Table 1), it seems plausible to suggest that this species may have developed good adaptability to various environments during its genomic evolution. The predicted CAZyme ORFs of the *P.* *taichungensis* I5 genome (highlighted in red in Fig. 2) were distributed throughout most contigs, although a few of the smaller contigs did not contain any CAZyme ORFs. A unique xanthan utilization gene cluster containing the ORFs for seven potential xanthan-degrading genes (labelled with their respective catalytic types and red stars in Fig. 2) was identified on the largest contig.

**Table 1:** General features of the genomes of *P. taichungensis* strains

No	Strain	Isolation source	Genome assembly	Size (Mbp)	GC content (%)	Protein-encoding ORFs*
1	53b	Soil	GCA_031583975.1	6.89	46.08	6,087
2	Cl17b	Gastrointestinal tract of gray wolf	GCA_047302525.1	6.94	46.30	6,064
3	NC1	Gold mine	GCA_003287275.1	6.99	46.18	6,126
4	NRS-1601	-	GCA_035803595.1	7.04	45.83	6,281
5	NRS-1605	-	GCA_035803535.1	7.05	45.85	6,245
6	179-I 5C2 NHS	Cleanroom floor	GCA_047246865.1	7.07	45.64	6,345
7	DB-4	Freshwater and sediment?	GCA_003201335.1	7.08	46.25	6,353
8	VTT E-133285	Low level radioactive waste repository experiment	GCA_002264305.1	7.08	46.15	6,278
9	NPDC087975	-	GCA_044742025.1	7.09	46.03	6,258
10	CIP110615T	-	GCA_965136425.1	7.10	45.85	6,330
11	NPDC055908	-	GCA_042270445.1	7.15	45.91	6,265
12	DSM 19942	Soil	GCA_013359905.1	7.23	45.71	6,303
13	BB507	Pine rhizosphere soil	GCA_046058935.1	7.33	45.83	6,296
14	I5 (VKM B-3510D)	A spoiled xanthan sample	In this study	7.57	45.53	6,936

^*^: Proteins were predicted by NCBI Prokaryotic Genome Annotation Pipeline (PGAP)

### Secretome features of strain *P. taichungensis* I5

Supernatant of cell cultures were collected by centrifugation and subjected to LC–MS/MS analysis as outlined in the Materials and methods section. Over all samples, 1,304 proteins of *P. taichungensis* I5 were identified in at least three replicates from one growth condition. As shown in supplementary Fig. S3a, a large set of proteins (1,300) were detected in XMM culture supernatants and fewer proteins in supernatants from GMM (85) and LB (124) cultures. However, principal component analysis (PCA, supplementary Fig. S3b) showed a clear clustering of the three biological replicates per growth condition. As it is hard to compare protein patterns in different growth conditions, and a gene cluster was found by genome analysis, we try to focus on this potential xanthan-degrading gene cluster by estimations of protein mass fractions using “intensity-based absolute quantification” (iBAQ) (Schwanhäusser et al. 2011). iBAQ values provide an absolute protein concentration estimate that can be used to compare the abundances of different proteins present in the same sample. By dividing the iBAQ value of a given protein through the summed iBAQ values of all proteins detected in a given sample, “protein mass fractions” were calculated for all proteins encoded in the xanthan gene cluster. As shown in supplementary Fig. S3c, all CAZymes putatively essential for xanthan depolymerization as well as other neighboring proteins were detected reproducibly in the XMM secretomes. Among them, GH88a (V4D07_03260) and xanthanase Pt_XanGH9 (V4D07_03230) were the most abundant, whereas GH88b (V4D07_03295) and xanthan lyase Pt_XanPL8b (V4D07_03310) were of low concentration. Of note, the ABC transport system including potential binding proteins (V4D07_03255, V4D07_03280) and permeases (V4D07_03245, V4D07_03250) was also detected reproducibly with significant protein mass fractions, indicating that they may play an important role for the transport of xanthan oligosaccharides into the cell. In addition, out of 13 GH3 and GH94 representatives (data not shown), only one GH3 (V4D07_32095) was detected in the secretome, which is encoded at a locus far away from the xanthan degradation gene cluster and shows 28.07% sequence similarity with a GH3 β-glucosidase from *B. intestinalis* (WP_118221772.1) and 24.44% with Mibgl3 from *Microbacterium* sp. XT11 (WP_067196459.1). This GH3 protein may cleave β-glucosidic linkages of cellobiose intracellularly generated from xanthan oligosaccharides.

### Characterization of recombinant xanthanolytic enzymes

The genes encoding the xanthanase and two xanthan lyase candidate enzymes were amplified via PCR and cloned in linearized expression vector pET24c as described in the Methods section for production of proteins with C-terminal His-tags. After confirmation of the correctness of plasmid construction, the fusion proteins were produced in *E.* *coli* Rosetta2 and purified by immobilized metal affinity chromatography. Analysis of recombinant xanthanase Pt_XanGH9, xanthan lyases Pt_XanPL8a and Pt_XanPL8b by 10% SDS-PAGE and western-blot demonstrated proteins with the expected sizes of about 132.4 kDa, 85.7 kDa and 98.4 kDa, respectively (supplementary Fig. S4). For Pt_XanGH9, multiple protein bands of lower molecular mass in addition to the 132.4 kDa band were observed. This is not unusual because similar to other known xanthanases (Han et al. 2024), this large enzyme has a modular structure which often leads to truncated proteins after recombinant expression.

Enzyme reactions were performed with variable amounts of enzyme in the presence of 5 mg mL^−1^ xanthan and assessed by quantification of the release of reducing sugar using the DNSA assay. The results indicated that all three enzymes were able to release reducing ends from native xanthan (supplementary Fig. S5a) although Pt_XanGH9 is expected to cleave the xanthan β-glucan backbone while Pt_XanPL8a and Pt_XanPL8b likely remove the terminal mannose from xanthan side chain. Considering the similar peak patterns observed with xanthanase Pt_XanGH9 and a xanthanase from *Paenibacillus nanensis* (Moroz et al. 2018) (see supplementary Fig. S5b), the cleavage site of xanthanase Pt_XanGH9 on the backbone of native xanthan is most likely at the reducing end of the branching glucose. Besides, the impact of terminal mannose on enzymatic activity was also investigated for xanthanase Pt_XanGH9 in reactions containing the same amount of substrate (native xanthan or XLT-xanthan) and enzyme. The TLC result suggested that this enzyme exhibited higher hydrolytic activity towards XLT-xanthan (obtained by xanthan lyase treatment of native xanthan) than towards native xanthan (see supplementary Fig. S1b). Of the two presumed xanthan lyases, which are expected to remove mannose residues from the side chains of native xanthan, Pt_XanPL8b appeared to release more reducing ends than Pt_XanPL8a. However, since the purity of the recombinant proteins was variable and optimized reaction conditions were not determined for these enzymes, these results merely enabled a qualitative estimation of substrate cleavage.

### Identification of degradation products

To clarify the degradation mechanism of *P. taichungensis* I5 xanthanase Pt_XanGH9, the degradation products released from native xanthan by Pt_XanGH9 were subjected to separation by preparative liquid chromatography (LC) using an acetonitrile/10% ammonium acetate gradient for elution and both ELSD and UV detectors for detection, as described in the Method section. Oligosaccharide products detected via ELSD and UV detector signals at different retention times (supplementary Fig. S6) were subjected to TOF–MS analysis and all measured in the negative electrospray ionization (ESI^−^) mode. Three types of xanthan pentasaccharides, namely acetylated-, diacetylated and pyruvylated pentamers with signals of m/z 883, 925 and 953 (Table 2 and supplementary Fig. S7) were only found after xanthanase Pt_XanGH9 treatment, indicating that Pt_XanGH9 cleaves the xanthan backbone at every second glucose residue, which is consistent with the cleavage preference of a xanthanase from *P.* *nanensis* (Moroz et al. 2018). The LC fractions containing xanthan pentasaccharides (fractions 16 and 17 in supplementary Fig. S6) were combined, dried and dissolved in Milli-Q water for further use as a substrate for xanthan degradation by the two xanthan lyases.

**Table 2:**
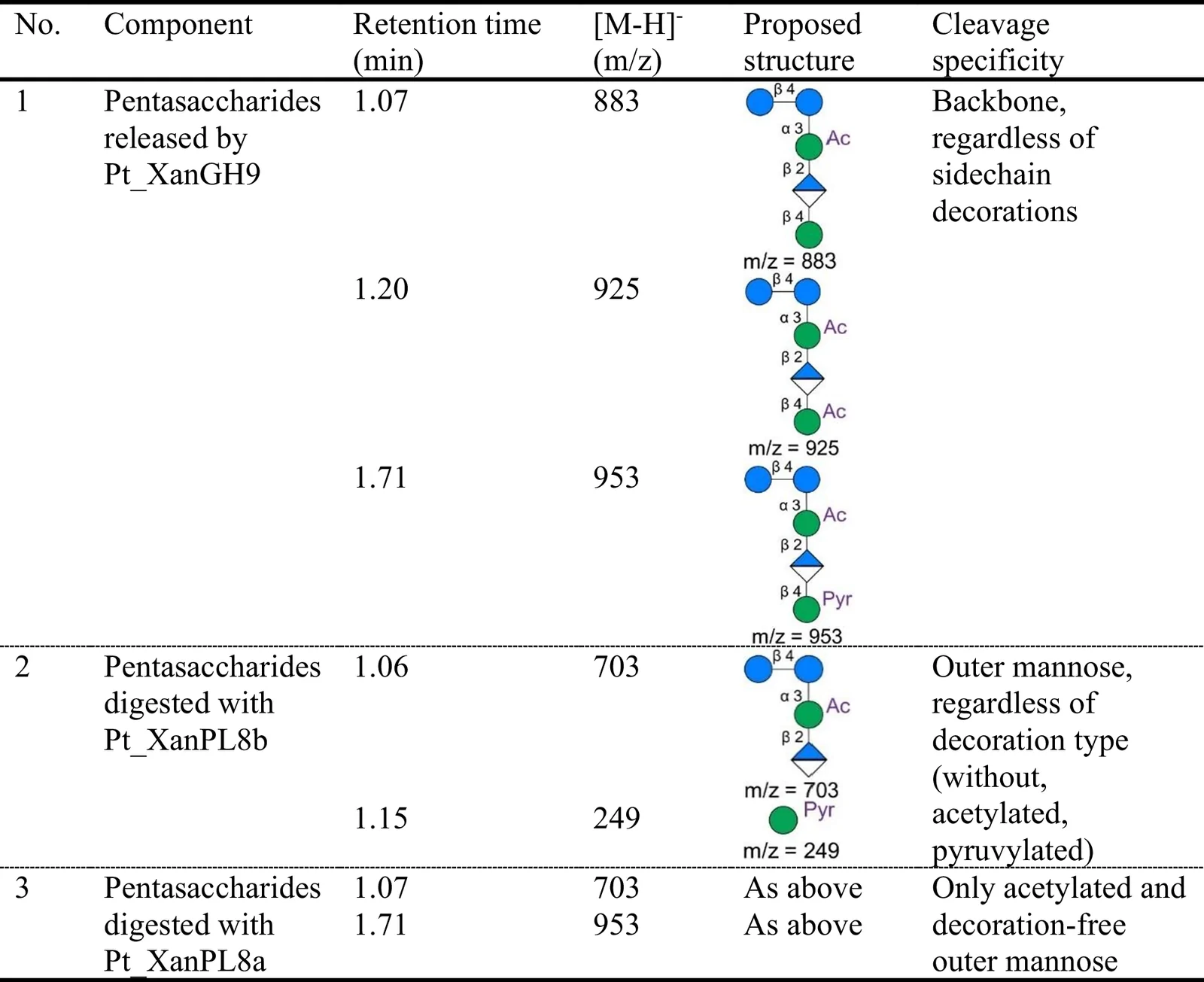
LC-MS analysis of products released from xanthan and xanthan-derived pentasaccharides

1. Products obtained by cleavage of native xanthan with *P. taichungensis* xanthanase Pt_XanGH9; 2. Products obtained after incubation of Pt_XanPL8b with the pentasaccharides purified after digestion of native xanthan with Pt_XanGH9(1.); 3. Products obtained after incubation of Pt_XanPL8a with the pentasaccharides purified after digestion of native xanthan with Pt_XanGH9 (1.)

Mannose Glucose Glucuronic acid or anhydro-glucuronic acid (when the residue is at the end after lyase cleavage). Ac: acetyl group, Pyr: pyruvyl group

Using xanthan-derived pentasaccharides, which have been purified by preparative LC, as the substrate, enzyme reactions (100 μL reaction mixtures containing 50 μL LC-purified pentasaccharides, 40 μL recombinant enzyme in 50 mM HEPES–NaOH buffer pH7.0) were performed to determine the functions of the two xanthan lyases Pt_XanPL8a and Pt_XanPL8b encoded in the *P. taichungensis* I5 xanthan degradation gene cluster. After 24 h incubation at 37 °C, digestion products were analysed by TOF–MS analysis. By treatment with Pt_XanPL8b, the signals corresponding to the three types of pentasaccharides (m/z of 883, 925 and 953 (ESI^−^); all acetylated at the inner mannose that is attached to the backbone glucose, see Table 2) were absent (top panel in supplementary Fig. S7) but instead, signals with an m/z of 703 and 249 were present (middle panel in supplementary Fig. S7), which represent the pentasaccharide cleavage products acetylated tetrasaccharide and pyruvylated mannose, respectively. In contrast, Pt_XanPL8a seems to be inactive on the pyruvylated pentasaccharide species since after incubation with this enzyme the signal at m/z 953 was retained but no signal was detected at m/z 249 (pyruvylated mannose) (bottom panel in supplementary Fig. S7). The disappearance of the other pentasaccharide signals at m/z 883 and 925 and the detection of a peak with m/z of 703 (acetylated tetrasaccharide) can be attributed to the loss of undecorated mannosyl and acetylated mannosyl residues, respectively. It can therefore be concluded that the two xanthan lyases Pt_XanPL8a and Pt_XanPL8b both cleave off terminal mannose residues from xanthan side chains (supplementary Fig. S5a) and xanthan-derived oligosaccharides but probably with different specificities. According to the absence or presence of an N-terminal signal peptide encoded in their respective genes, these enzymes presumably have different cellular localization (intracellular or extracellular, respectively).

## Discussion

### Pan-genome analysis within the species *P. taichungensis*

The genus *Paenibacillus* is well known for its ability to produce a variety of extracellular enzymes, such as cellulases, xylanases and pectinases (Grady et al. 2016). The potential for complex carbohydrate utilization is evident from the numbers of CAZymes encoded in the genomes of species from the *Paenibacillus* genus. For example, the genome of *Paenibacillus pabuli* strain E1 contains 372 genes for CAZymes, of which 98 are associated with the degradation of cellulose, hemicellulose, and pectin (Li et al. 2023). *P. taichungensis* BB507, a strain recently isolated from pine rhizosphere soil, has been predicted to possess 484 CAZyme genes in its genome (Ding et al. 2025). In this study, *P. taichungensis* strain I5 was found to have 275 genes encoding CAZymes, including 184 glycoside hydrolases (GH). Pan-genome analysis of 14 *P. taichungensis* strains (13 available in the NCBI database as of March, 2025, and strain I5 of this study; supplementary Table S3, Fig. 2a-d) and functional prediction of the core genes indicates that the ability to utilize and process carbohydrates is a fundamental and conserved trait in these organisms. Large numbers of genes encoding CAZymes and a high proportion of genes involved in carbohydrate uptake and metabolism may reflect adaptation to environments where carbohydrate availability represents a key selective pressure. The substantial proportion of metabolic genes in the accessory and unique gene pools suggests that differences in the strains’ metabolic capabilities significantly contribute to strain-to-strain variation. This diversity likely enables different strains to adapt to varying environmental conditions and nutrient sources by acquiring or losing metabolic functions of carbohydrate utilization. Interestingly, the xanthan utilization gene cluster of strain I5 is uniquely present in this strain but was not found in other strains with a threshold of 90% in the USEARCH algorithm.

### Functions encoded in the unique xanthan utilization gene cluster of *P. taichungensis* I5

Xanthan is a widely used natural polysaccharide produced by the bacterial species *Xanthomonas campestris* (García-Ochoa et al. 2000). Due to its complex chemical structure (a cellulose-like β-(1,4)-d-glucan backbone with substituted trisaccharide side chains) and an order–disorder conformation (single- or double-stranded helical structures depending on pH, ionic strength, temperature, and shear forces) in aqueous solution, it is recalcitrant to biodegradation in nature (Nsengiyumva and Alexandridis 2022). However, a number of specialized microorganisms have evolved the capacity to degrade and utilize this natural polymer. The enzymes encoded in the unique xanthan degradation gene cluster of *P. taichungensis* I5 share similarity with xanthan-degrading enzymes previously reported in *Bacillus* sp. GL1, suggesting that strain I5 may have a similar xanthan metabolism pathway. However, genes for mannosidase, xanthan lyase and unsaturated glucuronyl hydrolase are duplicated in this gene cluster of *P. taichungensis* I5. Strain I5 in addition possesses 11 GH3 and 2 GH94 enzymes encoded in other regions of the genome, which might be involved in cleaving β-glucosidic linkages of internalized oligosaccharides from xanthan decomposition (Gu et al. 2022; Ostrowski et al. 2022).

Proteome analysis comparing the secretome of *P. taichungensis* I5 grown in mineral media (GMM, XMM) and complex medium LB, resulted in a substantially larger number of proteins in XMM than in LB and GMM (supplementary Fig. S3a**)**. The reason for this large difference is not clear, but it seems likely that more of the XMM-grown cells had lysed than in the cultures grown in LB or GMM. All CAZyme proteins predicted to be encoded by the xanthanase gene cluster (see Fig. 3a) were uniquely identified in the XMM group by LC–MS/MS analysis (V4D07_03230, V4D07_03260, V4D07_03265, V4D07_03295, V4D07_03300, V4D07_03305 and V4D07_03310, see supplementary Table S2). Their similarity (24–62% sequence identity) with the amino acid sequences of previously characterized enzymes strongly suggest these enzymes play key roles in the xanthan degradation process. In addition, a β-glucosidase from the GH3 family (V4D07_32095) encoded far away from the xanthan degradation locus was detected in the culture when *P. taichungensis* I5 was grown in XMM. Possibly, this enzyme may contribute to xanthan utilization by cleaving the cellobiose moiety of internalized xanthan-derived oligosaccharides. A β-glucosidase from *Microbacterium* sp. XT11, Mibgl3, has been described to cleave the β−1,4 glucosidic bond of xanthan tetramer oligosaccharides [Glc-β1,4-(O3-Man-β1,2-GlcA)Glc] and also the β−1,2 glycosidic bond between the mannosyl and glucuronic acid residues in the side chains of xanthan [(-Glc-β1,4-(O3-Man-β1,2-GlcA-β1,4-Man)Glc-)_n_] (Gu et al. 2022).

Xanthan utilization loci have also been reported in *Microbacterium* sp. strain XT11, *B. intestinalis* and *Ruminococcaceae* strain UCG-13. In *Microbacterium* sp. strain XT11 it was found by quantitative real-time PCR that the expression of four enzymes belonging to the GH3, GH38, GH9, and PL8 families and one ABC transporter in the same cluster markedly increased when the strain was cultured in xanthan medium compared to glucose medium (Sun et al. 2019). Ostrowski et al. (2022) reported that the gut bacterium *Ruminococcaceae* strain UCG-13 possesses a xanthan degradation locus containing PL8, GH88, GH38, GH94, GH5 and CE (carbohydrate esterase) while in *B. intestinalis* an unknown CE, unknown PL, GH88, GH92 and GH3 are encoded in a cluster for xanthan depolymerization. Gene clusters dedicated to polysaccharide degradation, such as polysaccharide utilization loci (PULs) reported in Gram-negative *Bacteroidetes* bacteria, enable bacteria to efficiently break down complex carbohydrates into metabolizable sugars (Grondin et al. 2017). These clusters typically encode CAZymes, transporters, and regulatory proteins that work in concert to recognize, hydrolyze, and transport specific polysaccharides. In the *Bacillota* (formerly called *Firmicutes*) a Gram-positive PUL (gpPUL) was defined for a locus encoding at least one polysaccharide-degrading enzyme, a related carbohydrate transport system and a transcriptional regulator (Sheridan et al. 2016). *Roseburia intestinalis* L1-82 employs multiple clustered gene loci to degrade complex polysaccharides like xylan or β-mannan through tightly regulated enzymatic cascades (La Rosa et al. 2019; Leth et al. 2018). Such enzymatic cascades allow bacteria to release monosaccharides for further catabolism from natural polysaccharides, in the case of *P. taichungensis* I5 from xanthan. Further, potential gene clusters were also predicted using dbCAN3 CGC-Finder (Zheng et al. 2023). Together with the comprehensive annotation provided by eggNOG-mapper (Huerta-Cepas et al. 2019) and predicted 484 CAZyme loci by dbCAN2 (Zhang et al. 2018), 15 additional gene clusters were obtained after correction (see supplementary Table S4). For example, the gene cluster V4D07_05390–05415 encodes an extracellular GH76 (43.1% sequence similarity with characterized α−1,6-mannanase WP_037317868.1), an intracellular GH125 (52.3% sequence similarity with characterized exo-α−1,6-mannosidase BAB80132.1), a transcriptional regulator, and ABC transporter components including a substrate-binding protein and permeases. Given that GH76 enzymes function as endo-α-mannanases and GH125 enzymes as exo-1,6-α-mannosidases (Panwar et al. 2023), this cluster is likely involved in α−1,6-mannan degradation. Another example, cluster V4D07_18485–18525, contains two GH32 enzymes that may be responsible for fructan degradation. Collectively, these findings suggest that *P. taichungensis* I5 is a versatile polysaccharide utilizer.

In contrast to other typical xanthan degradation gene clusters, which generally include genes for four or five CAZymes involved in xanthan breakdown, the *P. taichungensis* I5 cluster uniquely features coding capacity for duplicated enzymes, including two xanthan lyases (Pt_XanPL8a and Pt_XanPL8b), two unsaturated glucuronyl hydrolases (GH88), and two mannosidases (GH38 and GH92). The redundancy of these enzymatic activities may offer several advantages for metabolizing complex substrates. Variants of enzymes may exhibit subtle differences in substrate specificity, enabling the bacteria to degrade a broader range of polysaccharide structures, i.e., in the case of *P. taichungensis* I5 other yet unknown polysaccharides similar to xanthan. In analogy, four characterized GH16 enzymes from a marine bacterium *Paraglaciecola hydrolytica* S66T have been shown to possess diverse functionalities: one enzyme hydrolyzes agarose, while the others target the substrate furcellaran. Among these, one is able to break down both β-carrageenan and κ/β-carrageenan, whereas the other two are specific for κ/β-carrageenan (Schultz-Johansen et al. 2018). Furthermore, enzyme variants may exhibit different properties with regard to their optimum pH, temperature or salt concentration, allowing the bacteria to effectively adapt to changing environments. It must be considered in this context that only the xanthanase, one of the xanthan lyases and possibly one of α-mannosidases are predicted to be extracellular (see supplementary Table S2) but the other enzymes were also detected in the culture supernatant to where they may have been released by partial cell lysis or a different mechanism.

Lyase-dependent xanthan degradation systems were reported for most known xanthan degraders, such as *P. nanensis* (Jensen et al. 2019) and *Paenibacillus alginolyticus* XL-1 (Ruijssenaars et al. 2000). In contrast, *Thermogutta terrifontis* apparently degrades xanthan based on a lyase-independent system, due to the absence of homologs of PL8 family lyases (Elcheninov et al. 2017). For *P. taichungensis* I5 we now report a twin-lyase-dependent system for xanthan degradation, where two xanthan lyases are produced that both can cleave native xanthan and xanthan-derived pentasaccharides. To date, the majority of characterized xanthan lyases are encoded with N-terminal signal peptides, suggesting that they function extracellularly. In accordance, xanthan lyase Pt_XanPL8b is synthesized as a precursor protein with an N-terminal signal peptide and thus probably is involved in the extracellular cleavage of native xanthan. On the other hand, due to its lack of a signal peptide, the main function of xanthan lyase Pt_XanPL8a may be to act on oligosaccharides intracellularly, although this enzyme was also detected in the culture supernatant by proteome analysis, as mentioned above. An intracellular localization is not commonly observed among known xanthan lyases (Jensen et al. 2019; Ruijssenaars et al. 2000; Yang et al. 2014). But there is a report from a xanthan lyase (30.6% sequence identity with Pt_XanPL8a) lacking a signal peptide from *Ruminococcaceae* strain UCG-13. This enzyme could only cleave xanthan pentasaccharides rather than polymeric xanthan (Ostrowski et al. 2022), while xanthan lyase Pt_XanPL8a from *P. taichungensis* I5 was active both on xanthan oligosaccharides and on polymeric xanthan. In addition, the activity of both Pt_XanPL8a and Pt_XanPL8b on xanthan-derived pentasaccharides suggests the existence of a tetramer and/or pentamer transporter system for the uptake of these oligosaccharides in *P. taichungensis* I5. Considering that xanthanase Pt_XanGH9 from *P. taichungensis* I5 can cleave native xanthan, albeit less efficiently than XLT-xanthan (see supplementary Fig. 1b), the initial step in xanthan degradation must not necessarily be xanthan lyase, which removes the terminal mannose residues leading to more accessibility for enzymes catalyzing subsequent cleavage reaction like in *Bacillus* sp. GL1 (Nankai et al. 1999). Pentasaccharides released from native xanthan by Pt_XanGH9 could be cleaved either outside (by extracellular xanthan lyase Pt_XanPL8b or some Pt_XanPL8a released from the cells, see above) or inside the cells (by xanthan lyase Pt_XanPL8a). The intracellular xanthan lyase cleavage requires the uptake of native xanthan-derived pentasaccharides into the cell as reported for tetrasaccharides in *Microbacterium* sp. XT11 (Sun et al. 2019). Intriguingly, there are two ORFs annotated to encode ABC transporter binding proteins within the xanthan degradation gene cluster. It is tempting to speculate that these binding proteins may have different binding specificities for different xanthan-derived oligosaccharides. It is also noteworthy that there are three pairs of consecutive ORFs, each for a histidine kinase and a response regulator, that appear to encode two-component regulatory systems. Although signals and target genes for these regulatory systems are currently unknown, this points to a complex transcriptional regulation of the degradation of xanthan and perhaps related polysaccharides.

### Activity of recombinant xanthanolytic enzymes

Recombinant enzyme production in *E. coli* of the *P. taichungensis* I5 xanthanase Pt_XanGH9 and the xanthan lyases Pt_XanPL8a and Pt_XanPL8b yielded protein preparations that allowed a preliminary characterization of the enzymes’ substrate cleavage properties. Enzyme reactions carried out at pH 7.0 and 37 °C using native xanthan as substrate revealed that xanthanase Pt_XanGH9 can cleave the native xanthan backbone, which has also been reported for xanthanases from a salt-tolerant bacterial culture HD1 (Hou Ching et al. 1986), *Microbacterium* sp. XT11 (Yang et al. 2019), *P. nanensis* (Moroz et al. 2018) and *Ruminococcaceae* strain UCG-13 (Ostrowski et al. 2022). Among these, *Microbacterium* sp. XT11 (51.1% sequence similarity with Pt_XanGH9) and *Ruminococcaceae* strain UCG-13 xanthanases hydrolyzed native and lyase-treated xanthan with comparable specificity, while *P. nanensis* xanthanase (51.5% sequence similarity with Pt_XanGH9) indicated more than 600-fold preference for the xanthan lyase-treated substrate as well as Pt_XanGH9. However, the difference in substrate preference observed for xanthanase Pt_XanGH9 in this study (see supplementary Fig. S1b) was not quantified. MS analysis indicated that three types of pentasaccharides (in Table 2) were produced from native xanthan by Pt_XanGH9, all with acetylated inner mannose, but with the terminal mannose either free of decorations, substituted with an acetyl group, or a pyruvyl group.

In addition to xanthanase, two xanthan lyases, Pt_XanPL8a and Pt_XanPL8b, which share 36.06% sequence similarity, were investigated for their activity on native xanthan as well as xanthan oligosaccharides. It was confirmed experimentally (see Table 2, supplementary Fig. S7) that they indeed are xanthan lyases. Using purified pentasaccharides (repeating unit of native xanthan) produced by xanthanase-treatment (Pt_XanGH9) as a substrate, extracellular Pt_XanPL8b was able to cleave off all terminal mannose moieties (either free of substituents, decorated with an acetyl group, or with a pyruvyl group) and produce tetrasaccharide (m/z 703) and pyruvylated mannose (m/z 249). The signal of pyruvylated mannose detected by MS is consistent with the observation that 50–70% terminal mannose residues are believed to be substituted with pyruvyl groups (Kool et al. 2014). Pt_XanPL8a on the other hand, which does not bear an N-terminal signal peptide, showed a different specificity and could only remove the acetylated and decoration-free mannose. The possession of two xanthan lyases, such as found with *P.* *taichungensis* I5 in this work is new among xanthan degraders. However, it is not rare that redundant enzymes from bacteria have evolved to target different substrates, such as two *Bacteroides ovatus* GH26 β-mannanases: one of the enzymes primarily produces mannobiose from mannan polysaccharides, while the other is outer membrane-associated and acts mainly on galactomannan producing relatively large oligosaccharide fragments (Bagenholm et al. 2017).

In summary, in this study we revealed for the first time a xanthan degradation system that utilizes two different xanthan lyases. A working model for xanthan utilization by *P.* *taichungensis* I5 is depicted in Fig. 3b: Growth in the presence of xanthan triggers expression of genes of the xanthan utilization gene cluster (Fig. 3a). This leads to the secretion of xanthanase Pt_XanGH9 and xanthan lyase Pt_XanPL8b, which results in the extracellular depolymerization of the backbone of xanthan into pentasaccharides (representing the repeating units of xanthan) and, via (partial) side chain de-mannosylation of polymeric xanthan and the pentasaccharides by action of the secreted xanthan lyase Pt_XanPL8b, also tetrasaccharides. The two types of oligosaccharides generated in this way are hypothesized to be captured by (different?) cytoplasmic membrane-bound substrate binding proteins (SBP) (see supplementary Table S2) and internalized into the cell by ABC transport. Pentasaccharides with acetylated and non-decorated mannose residues are thought to be cleaved into tetrasaccharides inside the cell by xanthan lyase Pt_XanPL8a. As described above, however, Pt_XanPL8a was also detected in the supernatant of cells growing exponentially in XMM medium. But since this enzyme is not synthesized with an N-terminal signal peptide, we assume its main role lies in intracellular degradation of internalized oligosaccharides. Thus, Pt_XanPL8a was drawn as an intracellular enzyme in Fig. 3b. We assume that the two GH88 enzymes encoded in the xanthan utilization gene cluster are responsible for the removal of glucuronic acid residues, and the GH92 or GH38 enzymes are expected to remove the inner mannose residues, perhaps with different specificity regarding acetylation and/or regarding the attachment of mannose to cellobiose or to glucose. Finally, a GH3 enzyme (not encoded in the gene cluster) is needed to cleave the β−1,4 glycosidic bond between the backbone glucose residues of oligosaccharides, thereby completing the enzyme repertoire required for full depolymerization of xanthan. To reveal the functions encoded in this gene cluster and to further elucidate the complete xanthan degradation process, the postulated roles of the mentioned intracellular enzymes in xanthan utilization await experimental verification by detailed enzyme characterization in the future. Besides, the deletion of this gene cluster and of selected individual ORFs therein from the chromosome would be an alternative way to draw a more detailed picture of their functions in xanthan utilization by *P. taichungensis* I5.

## Supplementary Information

Below is the link to the electronic supplementary material.

Supplementary file1 (PDF 990 KB)

## Data Availability

The mass spectrometric raw files as well as the MaxQuant output files have been deposited to the ProteomeXchange Consortium via the PRIDE partner repository and can be accessed using the identifier PXD068698 (https://proteomecentral.proteomexchange.org/cgi/GetDataset?ID = PXD068698, reviewer account username: reviewer_pxd068698@ebi.ac.uk, password: 45LlsoEDiVmB). The whole genome sequence of P. taichungensis strain I5 has been submitted to the NCBI database with the accession number JAZHOZ000000000.
