# Supplementary material for: A twin xanthan lyase-dependent xanthan degradation system in Paenibacillus taichungensis I5
Source: Appl Microbiol Biotechnol. 2026 Jan 22;110(1):31. doi: 10.1007/s00253-025-13684-y (PMC12827396; doi:10.1007/s00253-025-13684-y)
Supplement: Supplementary file 1 — Supplementary file1 (PDF 990 KB) [file 253_2025_13684_MOESM1_ESM.pdf]

Applied Microbiology and Biotechnology

Supplementary Materials for

A twin xanthan lyase-dependent xanthan degradation system in *Paenibacillus taichungensis* I5

Rui Han[1,2], Melanie Baudrexl[1], Oliver Frank[3], Christina Ludwig[4], Oksana V. Berezina[5], Sergey V. Rykov[5], Wolfgang Liebl[1*]

Affiliations:

1. Chair of Microbiology, School of Life Sciences, Technical University of Munich, Emil-Ramann-Str. 4, 85354 Freising, Germany
2. Michael Smith Laboratories, University of British Columbia, 2185 East Mall, Vancouver, BC V6T 1Z4, Canada
3. Chair of Food Chemistry and Molecular Sensory Science, School of Life Sciences, Technical University of Munich, Lise-Meitner-Straße 34, 85354 Freising, Germany
4. Bavarian Center for Biomolecular Mass Spectrometry (BayBioMS), School of Life Sciences, Technical University of Munich, Gregor-Mendel-Str. 4, 85354 Freising, Germany
5. National Research Centre “Kurchatov Institute”, Academician Kurchatov Sq. 1, 123182 Moscow, Russian Federation

*Corresponding Author:

Wolfgang Liebl, Email: wliebl@tum.de

Tel: +49 (8161) 71 5450, Fax: +49 (8161) 71 5475

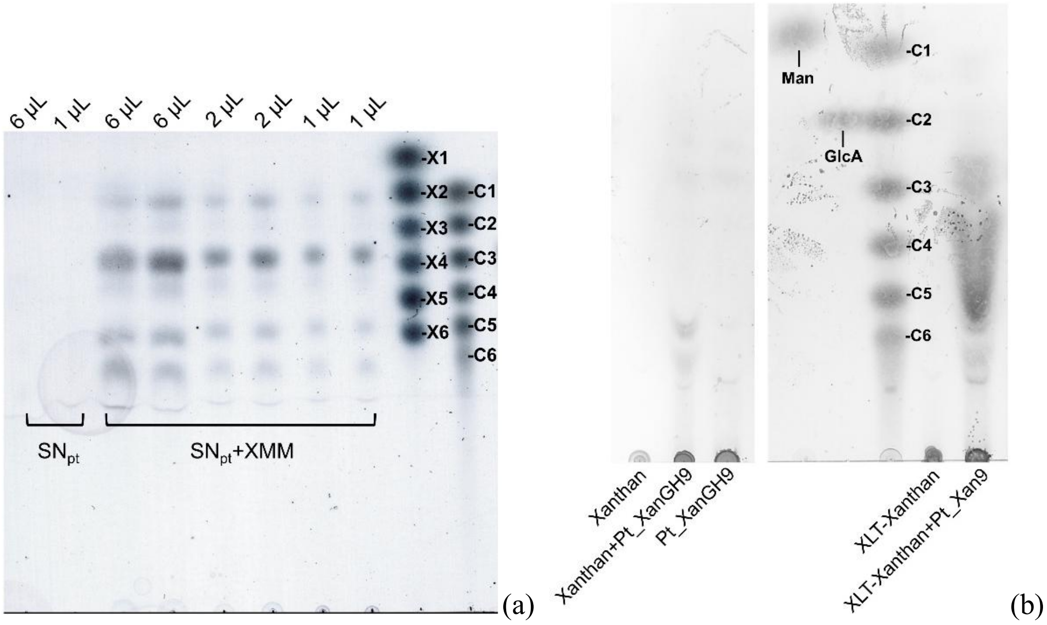

Fig. S1. Degradation products released from xanthan and XLT-xanthan by *P. taichungensis* I5 enzymes. (a) Sterile xanthan-containing XMM was incubated with cell-free culture supernatant of a XMM-grown culture of *P. taichungensis* I5. 500 µL of enzyme-containing cell-free culture supernatant (SN_{pt}) from *P. taichungensis* I5 grown in xanthan mineral medium (XMM) was incubated with same volume of fresh XMM at 37 ˚C for 24 h. Oligosaccharide standard mixtures were separated in the two lanes on the right. X1-X6: xylose, xylobiose, xylotriose, xylotetraose, xylopentaose, xylohexaose. C1-C6: glucose, cellobiose, cellotriose, cellotetraose, cellopentaose, cellohexaose. SN_{pt}: XMM supernatant after the growth of strain *P. taichungensis* I5, SN_{pt} + XMM: mixed enzymes collected from SN_{pt} were incubated with equal volume of XMM. (b) Xanthanase Pt_XanGH9 was incubated with 5 mg mL^{-1} xanthan and XLT-xanthan (with xanthan lyase pretreatment) in 100 µL reaction solution at 37 ˚C for 24 h. After denaturation and centrifugation, 15 µL supernatant was loaded on a silica gel 60 TLC plate. Man: mannose, GlcA: glucuronic acid.

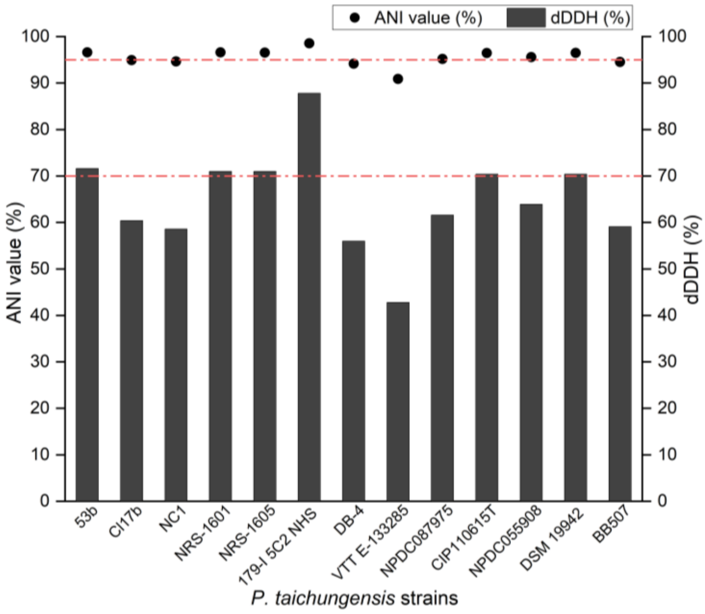

Fig. S2. Comparison of the strain I5 genome sequence with thirteen *P. taichungensis* genomes. Average nucleotide identity (ANI) and the digital DNA hybridization (dDDH) analysis was carried out with the genome sequence of strain I5 and thirteen *P. taichungensis* genome sequences retrieved from the NCBI database. The dashed red lines represent threshold values for species level relatedness of 95% for ANI and 70% for dDDH analysis. Strain information can be found in Table 1.

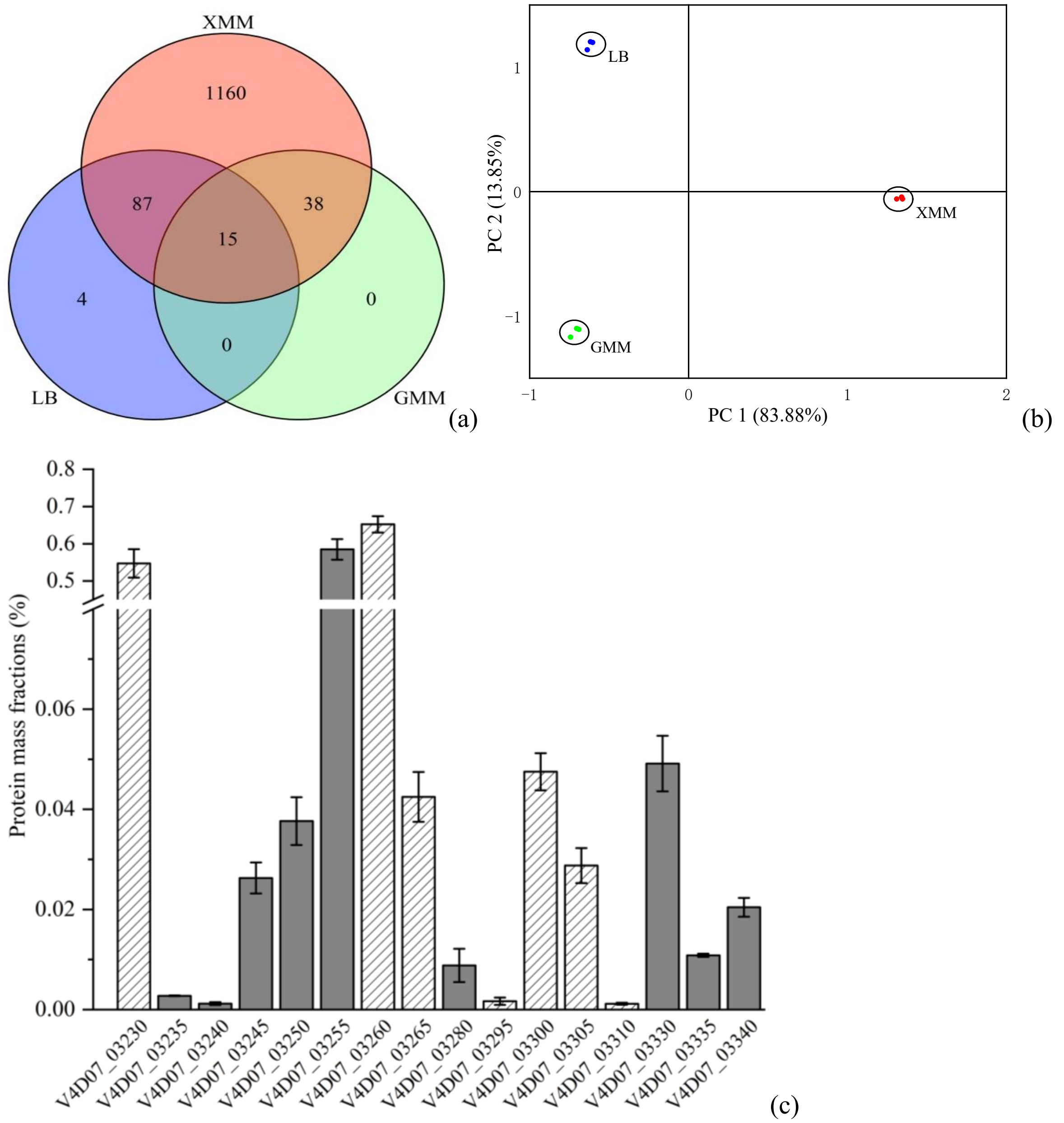

Fig. S3. Proteomic analysis of identified proteins secreted from *P. taichungensis* I5 after growth of three replicates in xanthan mineral medium (XMM), glucose mineral medium (GMM) and Lysogeny Broth (LB). (a) Venn diagram of identified proteins; (b) PCA analysis of identified proteins. (c) The mass fractions of the proteins encoded in the xanthan degradation gene cluster from *P. taichungensis* I5 detected in the culture supernatant after growth in the presence of xanthan. The hatched bars represent seven potential xanthan-degrading enzymes, including xanthanase Pt_XanGH9 (V4D07_03230), GH88a (V4D07_03260), GH92 (V4D07_03265), GH88b (V4D07_03295), xanthan lyase Pt_XanPL8a (V4D07_03300), GH38 (V4D07_03305) and xanthan lyase Pt_XanPL8b (V4D07_03310). Others may function as transporters or regulators of the xanthan metabolic pathway, related information can also be found in Fig. 3.

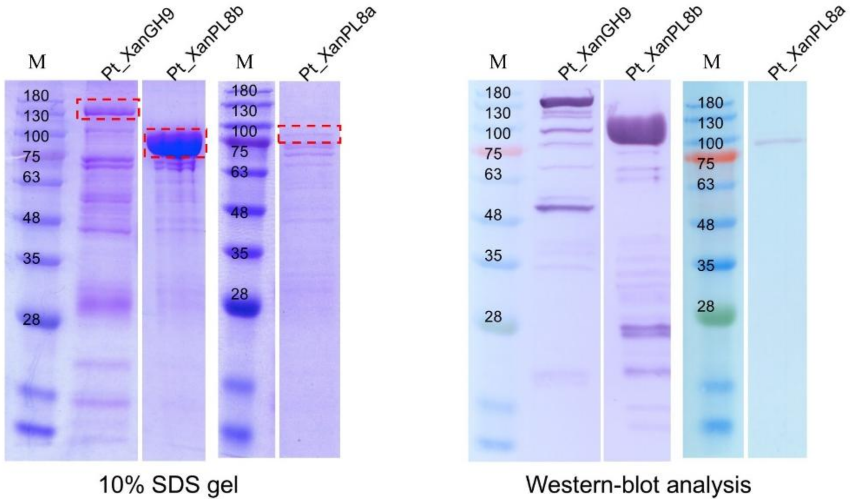

Fig. S4. Analysis of recombinant xanthan-degrading enzymes by 10% SDS-PAGE and Western blot. Eluate samples obtained from immobilized metal affinity chromatography were separated by 10% SDS-PAGE. The target proteins are marked with red boxes. M: BlueStar prestained protein marker (10–180 kDa), Pt_XanGH9: xanthanase, 132.4 kDa, Pt_XanPL8a: intracellular xanthan lyase, 85.7 kDa, Pt_XanPL8b: extracellular xanthan lyase, 98.4 kDa. The location of the encoding genes in the genome of *P. taichungensis* I5 can be found in Fig. 2.

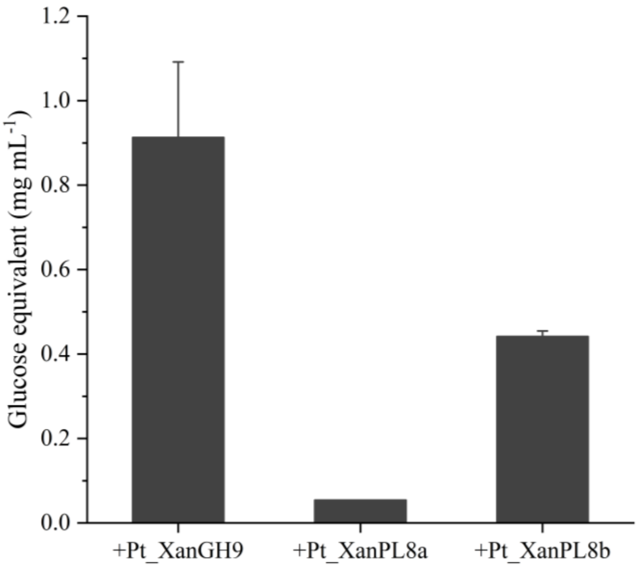

(a)

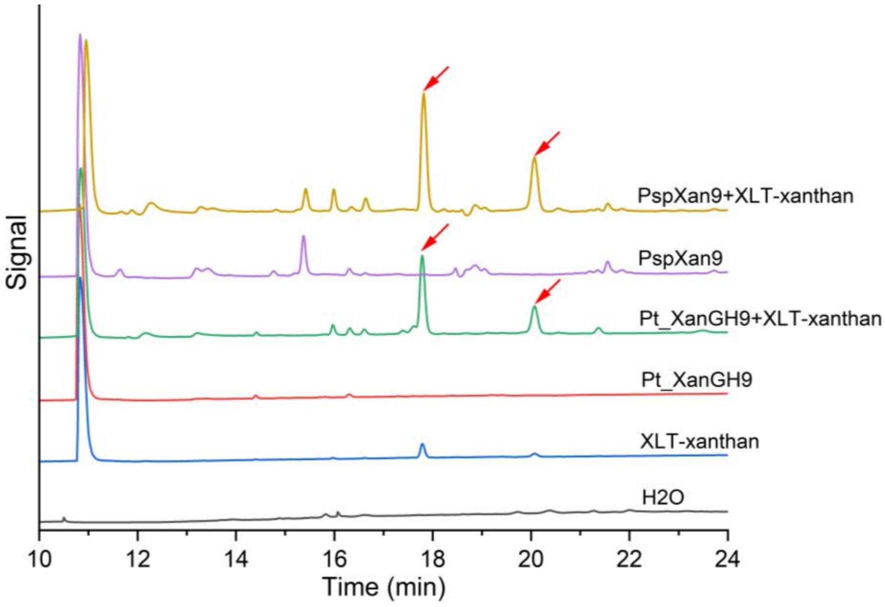

(b)

Fig. S5. Hydrolytic activity of recombinant *P. taichungensis* enzymes. (a) Reducing ends released from native xanthan by xanthanase Pt_XanGH9, xanthan lyases Pt_XanPL8a and Pt_XanPL8b. Reactions only containing native xanthan and buffer were used as controls. Except for Pt_XanPL8a, the assays were done in duplicates. (b) HPAEC-PAD analysis of degradation products from lyase-treated xanthan (XLT-xanthan) by xanthanase Pt_XanGH9 and *Paenibacillus nanensis Psp*Xan9 after 12 h incubation at 37 ˚C.

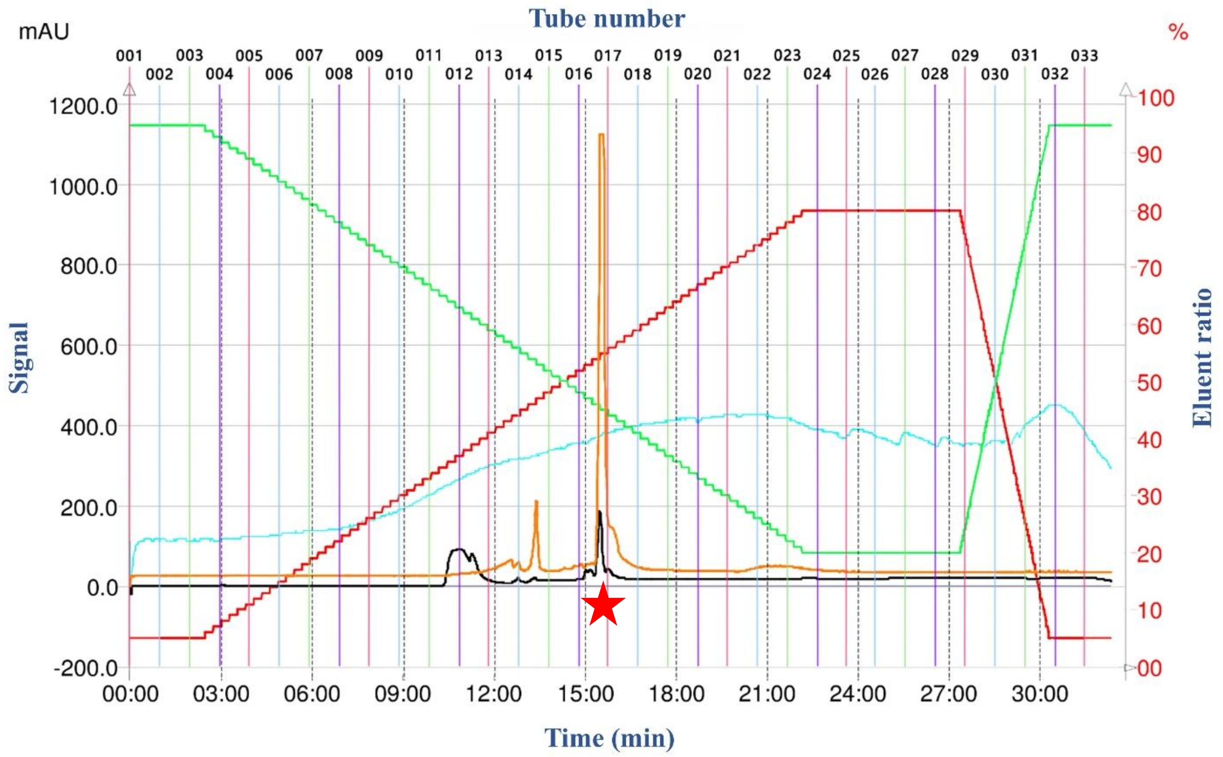

Fig. S6. Chromatogram of preparative LC for purification of oligosaccharides from xanthan digestion. Red line: percentage of 10% ammonium acetate; green line: percentage of acetonitrile; blue line: pressure; orange line: ELSD signal; black line: UV signal at 220 nm. Disparate peaks appearing with the different detectors can be attributed to different detection principles: while the UV detector only detects compound with suitable chromophores that absorb UV light, the ELSD detector responds to any non-volatile compound, regardless of its UV absorbance. Purified pentasaccharides were obtained from fractions 16 and 17 as indicated by a red star. Fraction numbers (001-033) are indicated at the top of the figure.

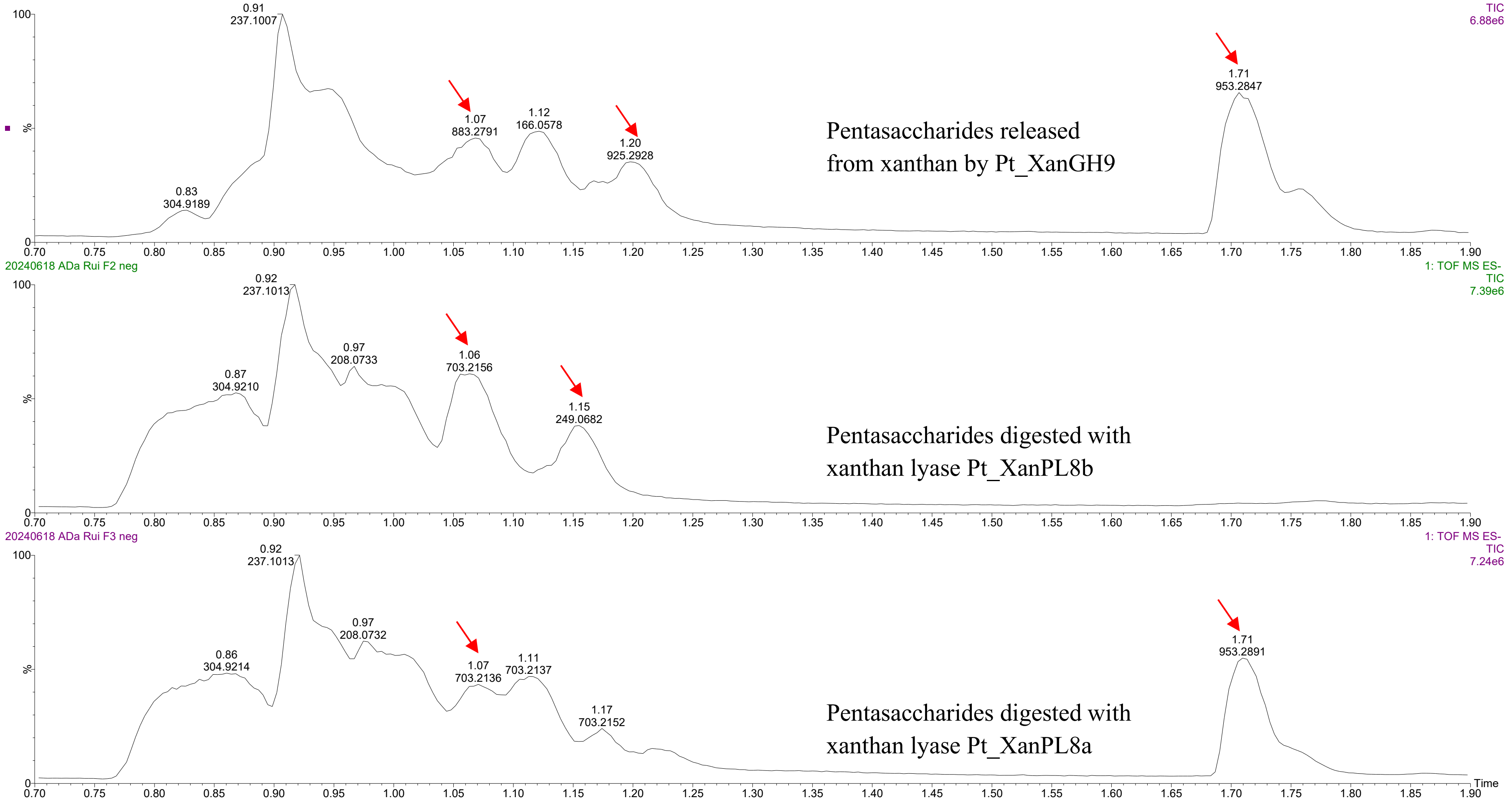

Fig. S7. TOF-MS analysis of pentasaccharide products released from native xanthan by recombinant *P. taichungensis* Pt_XanGH9 and of products of digestion of the pentasaccharide products by xanthan lyases Pt_XanPL8a and Pt_XanPL8b. Total ion chromatograms (TICs) of pentasaccharide-preparation from native xanthan by xanthanase Pt_XanGH9 treatment (top panel), and further digestion products by treatment with xanthan lyases Pt_XanPL8a (middle panel) and Pt_XanPL8b (bottom panel). The red arrows represent the signals of degradation products mentioned in Table 2.

Table S1. Primers used for genes from *P. taichungensis* I5 xanthan utilization locus.

Primer	Sequence (5‘-3‘)
Pt_XanGH9-F	CTTTAAGAAGGAGATATACAATGGTTACACCATTGCCAGGAC
Pt_XanGH9-R	AGTGGTGGTGGTGGTGGTGCTCAAGGCTGACTTCAGGAG
Pt_XanPL8a-F	CTTTAAGAAGGAGATATACAATGCTGAACTTAAAAAACAAA
Pt_XanPL8a-R	AGTGGTGGTGGTGGTGGTGCCCAAACTCAACTTGGAAC
Pt_XanPL8b-F	CTTTAAGAAGGAGATATACAATGGCGGATGAATACGATGGGAT
Pt_XanPL8b-R	AGTGGTGGTGGTGGTGGTGCGGGATACGCACAAATTTGAC

Table S2. The locus of a cluster of genes associated with xanthan degradation in the *P. taichungensis* I5 VKM B-3510D genome.

Locus tag by Prokka/Abbreviation	Annotation by Prokka	Predicted proteins from *Paenibacillus sonchi*	The closest characterized neighbors from other xanthan-degrading bacteria*	Locus tag by BPGA	Localization**
**00655/Pt_XanGH9**	Hypothetical protein	Glycoside hydrolase family 9 protein, endo-β-1,4-glucanase (xanthanase) (EC 3.2.1.-); WP_202676927.1; 98.68%	Xanthanase GH9 from *Paenibacillus nanensis*; AXR85426.1; 54.19% (Moroz et al. 2018)	V4D07_03230	Extracellular
**00656**	Regulator of RpoS	Response regulator; WP_039837101.1; 99.45%	-	V4D07_03235	Cytoplasmic
**00657**	Hypothetical protein	Sensor histidine kinase; WP_039837102.1; 99.31%	-	V4D07_03240	Cytoplasmic membrane
**00658**	Putative multiple-sugar transport system permease YteP	ABC transporter permease subunit; WP_202676926.1; 99.68%	-	V4D07_03245	Cytoplasmic membrane
**00659**	L-arabinose transport system permease protein AraQ	Carbohydrate ABC transporter permease; WP_039837103.1; 99.01%	-	V4D07_03250	Cytoplasmic membrane
**00660**	Hypothetical protein	ABC transporter substrate-binding protein; WP_039837104.1; 99.24%	-	V4D07_03255	Cytoplasmic membrane
**00661/GH88a**	Unsaturated glucuronyl hydrolase	Glycoside hydrolase family 88 protein; WP_039837106.1; 99.73%	Unsaturated glucuronyl hydrolase GH88 from *Bacillus* sp. GL1;	V4D07_03260	Cytoplasmic

**00662/GH92**	Hypothetical protein	Glycosyl hydrolase family 92; WP_039837107.1; 99.03%	BAA84216.1; 39.28% (Hashimoto et al. 1999) α-1,2-Mannosidase GH92 from *Microbacterium* sp. M-90; BAA76709.1; 23.93% (Nakajima et al. 2000)	V4D07_03265	Extracellular?
00663	Putative multiple-sugar transport system permease YteP	Sugar ABC transporter permease; WP_039837109.1; 99.68%	-	V4D07_03270	Cytoplasmic membrane
00664	Hypothetical protein	Carbohydrate ABC transporter permease; WP_039837110.1; 99.66%	-	V4D07_03275	Cytoplasmic membrane
**00665**	Hypothetical protein	ABC transporter substrate-binding protein; WP_039837112.1; 99.41%;	-	V4D07_03280	Cytoplasmic membrane
00666	Hypothetical protein	Histidine kinase; WP_167330830.1; 99.14%	-	V4D07_03285	Cytoplasmic membrane
00667	Regulator of RpoS	Response regulator; WP_051052022.1; 99.07%	-	V4D07_03290	Cytoplasmic
**00668/GH88b**	Unsaturated chondroitin disaccharide hydrolase	Glycoside hydrolase family 88 Protein; WP_051052023.1; 98.39%	Unsaturated glucuronyl hydrolase GH88 from *Bacillus* sp. GL1; BAA84216.1; 45.81% (Hashimoto et al. 1999)	V4D07_03295	Cytoplasmic
**00669/Pt_XanPL8a**	Xanthan lyase	Polysaccharide lyase 8 family protein; WP_039837115.1; 93.19%	Xanthan lyase XalA from *Paenibacillus alginolyticus*;	V4D07_03300	Cytoplasmic

**00670/GH38**	Hypothetical protein	α-Mannosidase; WP_039837116.1; 99.05%	AAG24953.1; 36.27% (Ruijssenaars et al. 1999) α-D-Mannosidase GH38 from *Bacillus* sp. GL1; BAB40444.2; 62.48% (Nankai et al. 2002)	V4D07_03305	Cytoplasmic
**00671/Pt_XanPL8b**	Xanthan lyase	Polysaccharide lyase β-sandwich domain-containing protein; WP_051052025.1; 100.00%	Xanthan lyase XalA from *P. alginolyticus*; AAG24953.1; 54.13% (Ruijssenaars et al. 1999)	V4D07_03310	Extracellular
00672	Hypothetical protein	Sensor histidine kinase; WP_051052027.1; 99.83%	-	V4D07_03315	Cytoplasmic membrane
00673	Putative response regulatory protein	Response regulator; WP_039837101.1; 99.45%	-	V4D07_03320	Cytoplasmic
00674	Hypothetical protein	Sensor histidine kinase; WP_039837102.1; 99.31%	-	V4D07_03325	Cytoplasmic membrane

*% means sequence identity identified by blastP.

**The localization was predicted by DeepLocPro-1.0 (Moreno et al. 2024).

Locus tags in bold represent proteins detected in the cell-free supernatant of xanthan mineral medium during the bacterial growth by LC-MS/MS analysis. The relative abundances can be found in supplementary Fig. S3.

Table S3. Pan-genome analysis for the species *P. taichungensis*.

Strain	Core genes	Accessory genes	Unique genes	Exclusively absent genes
VTT E-133285	3655	1099	1500	373
DB-4	3655	1677	1003	125
NC1	3655	2134	299	9
DSM 19942	3655	2536	82	5
53b	3655	2165	254	2
NRS-1605	3655	2558	5	0
NRS-1601	3655	2570	19	0
NPDC055908	3655	2091	504	17
NPDC087975	3655	1973	618	27
BB507	3655	2222	405	3
179-I 5C2 NHS	3655	2272	404	7
Cl17b	3655	1922	475	28
CIP110615T	3655	2528	129	13
I5 (VKM B-3510D)	3655	2286	974	16

Core genes are defined as those present in all eight genomes, whereas unique genes are those present exclusively in one genome. Accessory genes are those present in two to seven genomes, whereas genes that are present in one or more genomes but completely absent in the current genomes under analysis are referred to 'exclusively absent genes'.

Table S4. Predicted gene clusters in *P. taichungensis* strain I5.

Potential gene cluster	CAZymes	Transport system*	Transcriptional regulator**	Locus tag by BPGA (V4D07_ + number)
1	GH38, GH85+CBM32	ABC	HK	00925–00955
2	GH9, 2x GH88, GH92, GH38, 2x PL8	2X ABC	3x HK, 2x AraC	03230–03335
3	GH76, GH125	ABC	HK	05390–05415
4	GH88, PL29?+CBM32	ABC	AraC	06575–06605
5	GH28+CBM32, GH106, GH130	ABC	HK, AraC–CheY	07735–07770
6	GH130, GH125	ABC	2x AraC, HK	07875–07915
7	GH26+CBM35+CBM3, GH29, GH123	ABC	2x TetR	08875–08935
8	GH13_2+CBM20, GH13_20+CBM34, GH2+CBM6	ABC, MFS	LacI	09915–09975
9	GH9+CBM3, GH48+CBM3, GH51, GH29, CBM9, GH18	ABC	LytR_cpsA_psr	11065–11165
10	GH5, GH3+CBM6	_	LacI, HK	18295–18335

11	GH32+CBM66, GH32	ABC	LacI	18485–18525
12	2x GH18+CBM12, CE8, GT2, GH126	ABC, MFS	AraC	19455–19500
13	GH27, GH110, 3x GH95, 2x GH36, GH36+CBM35	2x ABC	HK, 2x AraC	21615–21705
14	GH85, GH3, 2x GH38, GH125	ABC	HK	27150–27210
15	GH1, GH95	ABC	HK, AraC-CheY	30655–30695
16	GH88, GT2, GT1, CE4	ABC	AraC	32170–32220

*: Transporters mainly include major facilitator superfamily (MFS) and ATP-Binding Cassette (ABC) transporters.
**: Transcriptional regulators mainly include histidine kinase (HK), lactose operon (LacI), L-arabinose operon (AraC), CheY-like receiver domain (AraC-CheY), tetracycline resistance genes (TetR) and LytR_cpsA_psr.
Gene clusters were initially predicted using dbCAN3 CGC-Finder (Zheng et al. 2023) and then refined with additional annotation information from eggNOG-mapper (Huerta-Cepas et al. 2019) and CAZyme analyses.

Table S5. COG distribution of *P. taichungensis* pan-genome analysis.

Code	COG CATEGORY	Core	Accessory	Unique
[D]	Cell cycle control, cell division, chromosome partitioning	0.81	0.47	0.52
[M]	Cell wall/membrane/envelope biogenesis	3.73	5.42	6.00
[N]	Cell motility	1.32	1.60	2.27
[O]	Post-translational modification, protein turnover, and chaperones	2.86	1.72	2.27
[T]	Signal transduction mechanisms	6.37	6.54	6.77
[U]	Intracellular trafficking, secretion, and vesicular transport	0.98	1.15	2.06
[V]	Defense mechanisms	1.60	2.78	2.51
[J]	Translation, ribosomal structure and biogenesis	4.94	1.30	1.26
[K]	Transcription	10.97	14.48	13.40
[L]	Replication, recombination and repair	3.17	3.73	8.03
[C]	Energy production and conversion	3.87	2.90	2.58
[G]	Carbohydrate transport and metabolism	12.96	13.59	9.53
[E]	Amino acid transport and metabolism	9.14	6.99	5.48
[F]	Nucleotide transport and metabolism	2.55	1.10	1.19
[H]	Coenzyme transport and metabolism	3.06	1.57	1.33

[I]	Lipid transport and metabolism	2.36	2.61	2.90
[Q]	Secondary metabolites biosynthesis, transport, and catabolism	2.08	3.20	3.87
[P]	Inorganic ion transport and metabolism	6.40	6.60	4.68
[R]	General function prediction only	14.00	15.66	15.74
[S]	Function unknown	6.84	6.57	7.61

All COG categories match the order presented in Fig.2c.

Table S6. KEGG distribution of *P. taichungensis* pan-genome analysis.

KEGG CATEGORY	KEGG SUB-CATEGORY	Core	Accessory	Unique
Metabolism	Amino acid metabolism	9.69	6.14	5.65
	Biosynthesis of other secondary metabolites	1.21	1.42	1.62
	**Carbohydrate metabolism**	**14.58**	**12.58**	**11.87**
	Energy metabolism	5.15	4.12	3.23
	Glycan biosynthesis and metabolism	2.08	2.47	1.94
	Lipid metabolism	4.15	4.64	4.68
	Metabolism of cofactors and vitamins	5.58	4.94	3.96
	Metabolism of other amino acids	2.77	1.80	1.05
	Metabolism of terpenoids and polyketides	1.30	1.72	1.70
	Nucleotide metabolism	5.37	4.57	3.80
	Overview	11.03	6.29	7.27
	Xenobiotics biodegradation and metabolism	1.56	2.40	3.15
Environmental_Information_Processing	**Membrane transport**	**11.64**	**18.43**	**10.50**
	Signal transduction	6.53	10.11	9.69
	Signaling molecules and interaction	0.00	0.67	1.37

Genetic_Information_Processing	Folding sorting and degradation	1.95	0.52	0.89
	Replication and repair	3.16	3.60	7.19
	Transcription	0.22	0.07	0.08
	Translation	3.94	0.37	0.73
Human_Diseases	Cancers	1.00	1.27	1.78
	Drug resistance	1.30	1.72	1.86
	Endocrine and metabolic diseases	0.13	0.00	0.08
	Immune diseases	0.09	0.07	0.08
	Infectious diseases	0.52	2.02	3.96
	Neurodegenerative diseases	0.17	0.60	0.16
	Substance dependence	0.00	0.22	0.24
Cellular_Processes	Cell growth and death	0.56	0.67	0.97
	Cell motility	2.21	2.55	2.58
	Cellular commiunity	0.00	0.67	1.53
	Transport and catabolism	0.30	0.82	0.57
Organismal_Systems	Digestive system	0.17	1.05	2.02
	Endocrine system	0.91	0.52	1.21

Environmental adaptation	0.17	0.22	0.89
Excretory system	0.04	0.00	0.00
Immune system	0.17	0.52	1.37
Nervous system	0.35	0.15	0.24
Sensory system	0.00	0.00	0.08

All KEGG subcategories match the order presented in Fig.2d. Core genes show enrichment in ‘Carbohydrate metabolism’ (in bold), whereas accessory genes are enriched in ‘Membrane transport’ (in bold).
